# Breaking the Structure of Liquid Hydrogenated Alcohols Using Perfluorinated *tert*-Butanol: A Multitechnique Approach (Infrared, Raman, and X-ray Scattering) Analyzed by DFT and Molecular Dynamics Calculations

**DOI:** 10.1021/acs.jpcb.1c10776

**Published:** 2022-03-01

**Authors:** M. Isabel Cabaço, Marcel Besnard, Carlos Cruz, Pedro Morgado, Gonçalo M. C. Silva, Eduardo J. M. Filipe, João A. P. Coutinho, Yann Danten

**Affiliations:** † CeFEMA, Centro de Física e Engenharia de Materiais Avançados, Departamento de Física, Instituto Superior Técnico, Universidade de Lisboa, 1049-001 Lisboa, Portugal; ‡ Laboratory of Instrumentation, Biomedical Engineering and Radiation Physics (LIBPhys-UNL), Department of Physics, NOVA School of Science and Technology, NOVA University Lisbon, 2829-516 Caparica, Portugal; § GSM Institut des Sciences Moléculaires, CNRS (UMR 5255), Université Bordeaux I, 351, Cours de la Libération, 33405 Talence Cedex, France; ∥ Centro de Química Estrutural, Instituto Superior Técnico, Universidade de Lisboa, Av. Rovisco Pais 1, 1049-001 Lisboa, Portugal; ⊥ CICECO, Departamento de Química, Universidade de Aveiro, 3810-193 Aveiro, Portugal

## Abstract

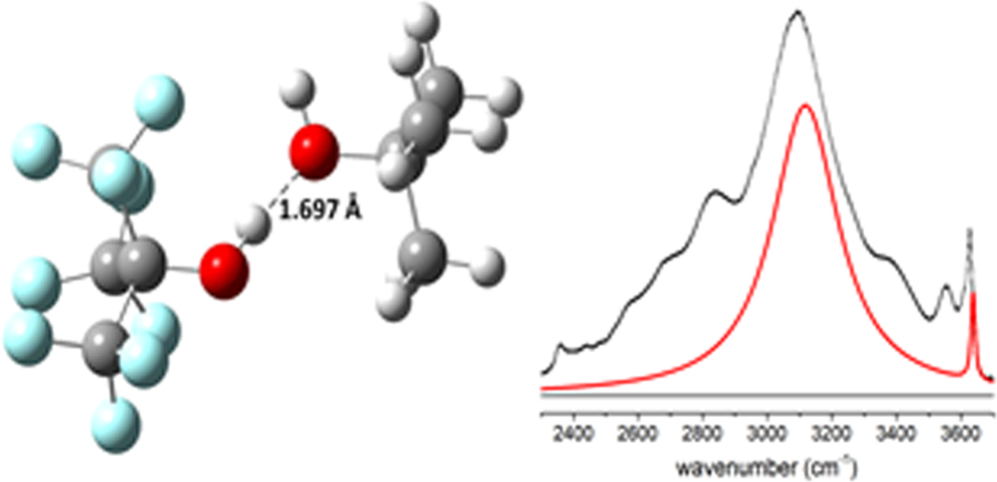

The state of aggregation at room temperature of *tert*-butanol (TBH) and perfluoro *tert*-butanol (TBF) liquid mixtures is assessed by vibrational spectroscopy (Raman and infrared) and X-ray diffraction and analyzed using density functional theory (DFT) and molecular dynamics (MD) simulations. It is shown that larger clusters (mostly tetramers) of TBH are destroyed upon dilution with TBF. Small oligomers, monomers, and mainly heterodimers are present at the equimolar concentration. At variance with slightly interacting solvents, the signature of hetero-oligomers is shown by the appearance of a new broad band detected in the infrared region. The same spectral observation is detected for mixtures of other hydrogenated alcohols (methanol and 1-butanol). The new infrared feature is unaffected by dilution in a polar solvent (CDCl_3_) in a high-concentration domain, allowing us to assign it to the signature of small hetero-oligomers. MD simulations are used to assess the nature of the species present in the mixture (monomers and small hetero-oligomers) and to follow the evolution of their population upon the dilution. Combining MD simulations with DFT calculations, the infrared spectral profile is successfully analyzed in equimolecular mixtures. This study shows that TBF is a structure breaker of hydrogen-bonded alcohol networks and that the TBF (donor)–TBH (acceptor) heterodimer is the dominant species in an extended range of concentration, centered in the vicinity of the equimolar fraction.

## 1 Introduction

Neat liquid *tert*-butanol (TBH) and its solutions in “inert” or weakly interactive solvents have been extensively studied experimentally (infrared, Raman, X-ray, and neutron diffraction, NMR)^1−26^ and theoretically.^5,9,10,16−20,27−32^ In the pure liquid at room temperature, it was shown that species consisting of monomers, dimers, cyclic tetramers, and hexamers coexist. Among the aggregates, the tetramer population was found, by far, to be the dominant one with a proportion of about 65%.^11−13^ Neutron and X-ray scattering studies led to the conclusion that TBH forms reverse micelle-like aggregates in which the OH groups of the molecules involved in hydrogen bonding form a core, surrounded by the methyl groups facing outward.^17,18,21^ Moreover, spatial correlations between neighboring clusters have been demonstrated by neutron diffraction by the existence of a pre-peak in the static structure factor.^13^ Molecular dynamics simulation results agree with the formation of these small reverse micelles of about four to six molecules (cyclic hexamer).^16,31,32^ In summary, all of these studies indicate that pure TBH presents a rather structured liquid phase with a predominance of hydrogen-bonded molecules forming reverse micelle-like structures, involving at least four to six molecules.

Studies of TBH diluted in inert solvents allowed, following the progressive disappearance of larger aggregates observed in concentrated solutions, the concomitant formation of smaller species as the solvent concentration increased in the solution. Thus, valuable information about the evolution of the speciation in the mixture from the pure liquid alcohol to the very diluted mixture are available.

In previous work, we have studied systems with coexisting hydrogenated and fluorinated chains, exploring the tendency of these mutually phobic segments to segregate and how this tendency affects the liquid properties and induces organization.^33,34^ We have focused on systems where hydrogenated and fluorinated moieties are joined by some form of mutual association,^35−43^ hoping to clarify how this affects the structure of the liquid and induces reorganization. In particular, we have directed our attention to mixtures of hydrogenated and fluorinated alcohols, in which hydrogen bonds can be seen as on–off associative interactions between the two phobic components.^44,45^ A strategy combining thermodynamics, spectroscopy, and molecular dynamics (MD) simulations was used. Evidence was found for the existence of a balance between hydrogen bonding (both homo and hetero) and the unfavorable dispersion forces between the hydrogenated and fluorinated chains. In terms of liquid structure, simulation and infrared spectroscopy results suggest a segregation effect between hydrogenated and fluorinated chains. In addition, the presence of fluorinated groups was demonstrated to induce conformational changes in the hydrogenated chains, from the usually preferred all-trans to more globular arrangements involving gauche conformations. This new coiling effect was also detected in mixtures of alkanes and perfluoroalkanes.^46^

It is in this context that we have focused our attention to perfluorinated *tert*-butanol (TBF). This molecule has been studied in the gaseous phase, in rare gas matrices and in the liquid state.^47−54^ Recent investigations combining several techniques (vibrational spectroscopy and X-ray diffraction) analyzed at the light of quantum density functional theory (DFT) and molecular dynamics have confirmed that this alcohol is poorly associated.^55^ In particular, it was found that the liquid phase is essentially constituted by monomers, with a population of about 70%, accompanied by a minor fraction of dimers and almost negligible trimers. It was shown that this weak tendency for self-association is mostly due to the electron-withdrawing effect of its CF_3_ groups.^56^ This result allows inferring that TBF will be an excellent candidate as a proton donor in the experimental studies of hydrogen-bonded systems because of its strong acidic properties. This is also reflected in the low value of its p*K*_a_ = 5.4^57^ compared to those of TBH or usual alcohols (methanol, octanol) having a pKa typically close to 19.^58^ A final advantage of TBF arises also from the larger volume of the CF_3_ groups, compared to that of CH_3_, leading to a large stereochemical hindrance preventing the formation of sequential hydrogen bonding and aggregation beyond the dimer. Finally, the TBF molecule is globular, thus avoiding the complication due to chain conformations existing with linear molecules as mentioned above. In view of all of these considerations, it appears of great interest to study binary mixtures of TBF with hydrogenated alcohols as it is expected that the acidic hydroxyl group of TBF should strongly interact with the more basic OH group of the alcohols.

Very recently, we have shown that in the gas phase at room temperature, TBF easily forms hydrogen-bonded heterodimers with hydrogenated alcohols (TBH, CH_3_OH, and 1-butanol).^59^ Therefore, we anticipate that in the liquid phase, the destruction of clusters of TBH by TBF should be concomitantly accompanied by an increasing formation of small-sized fluorinated–hydrogenated heteroclusters, as the amount of TBF is continuously increased in the mixture. Such breaking of a hydrogenated alcohol network by a monomeric population of fluoro-alcohols having a strong acidic character would constitute a new result. Incidentally, we emphasize that only a limited number of studies have been performed on mixtures of fluoro-alcohol with bases containing oxygen.^48−51^ There is a single study in which the exceptional H-bond donor character of the TBF molecule has been used in the context of molecular recognition with strong H-bond acceptors other than TBH.^60^

It is in this context and based upon the previous considerations that we have decided to study the mixtures of TBH and perfluorinated *tert*-butanol (TBF) using an experimental multitechnique approach based on vibrational spectroscopy (infrared and Raman) and X-ray scattering. Further support by the analysis of quantum DFT calculations and molecular dynamics simulations is also reported.

## 2 Experimental Conditions

TBH, methanol, and 1-butanol were obtained from Sigma-Aldrich and TBF from Apollo Scientific (purity greater than 99%), as well as CD_3_OH and *tert*-butanol-D9 (CD_3_)_3_COH (TB-D9) from Eurisotop (purity 98%) were dried using molecular sieves (3 Å). CDCl_3_ from Eurisotop (purity 99.8%) was used as a solvent. The water content was measured by Karl-Fisher titration and found to be 400 ppm.

The Raman spectra were measured with a resolution of 4 cm^–1^ in the spectral range 200–3800 cm^–1^ on a Horiba Jobin-Yvon XploRA spectrometer in a back-scattering geometry after collecting for 20 s and accumulated 60 times.

The infrared spectra were measured on a Bruker-Alpha FT-IR spectrometer with a 4 cm^–1^ resolution in the spectral range 400–4000 cm^–1^ after collecting 64 scans.

X-ray diffraction measurements were performed using Cu Kα radiation, allowing us to record patterns on a range of momentum transfer *Q* from 0.15 to 2 Å^–1^. All of the experiments were performed at 298 K. Experimental details are given in the Supporting Information (SI).

The structure, spectral features, and stabilization energy of the different aggregates have been assessed by DFT calculations using the program Gaussian16.C01 package.^61^ The details of the calculations are reported in the SI.

Molecular dynamics simulations were performed using atomistic molecular models of TBH and TBF based on the OPLS-AA force field.^62^ These models, which include new partial charges derived from *ab initio* calculations, are described in detail in a previous work (Table S1).^55^ To account for the peculiar weak interactions between fluorinated and hydrogenated moieties, the crossed Lennard-Jones parameters between the fluorine atoms of TBF and the methyl hydrogen atoms of TBH were modified from the combining rule values, as proposed for [*n*-butanol + 2,2,3,3,4,4,4-heptafluor-1-butanol] mixtures in a previous work^45^ (cross-interaction energy reduced by 20% and cross-interaction size increased by 3.5%). The GROMACS 5.0.7 simulation package^63^ was used to run and analyze the simulations, and the X-ray diffraction spectra were calculated from the simulation trajectories by the TRAVIS software.^64^ Detailed descriptions of the molecular models used and of the simulation procedures are given in the SI.

## 3 Experimental Results

### 3.1 Raman and Infrared Spectra of Neat TBH and TBF Alcohols

#### 3.1.1 TBH

**Figure 1:**
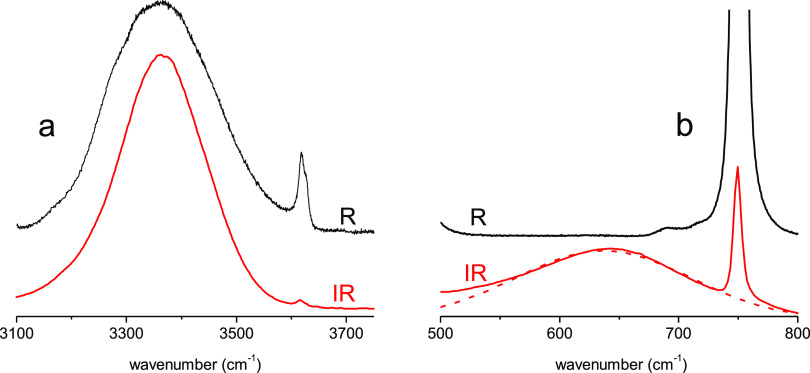
Raman (black) and Infrared (red) spectra of pure TBH in the spectral domain of: (a) ν_OH_-stretching vibration, (b) γ_OH_ out-of-plane vibration; fitted Gaussian profile to IR spectrum (---).

The Raman and infrared spectra of the ν_OH_ stretching vibration of liquid TBH are displayed in Figure 1a. The Raman spectra were corrected from CH combination bands using deuterated *tert*-butanol TB-D9 (Figure S1). These spectra present a weak composite band observed at a high frequency (*ca.* 3616 cm^–1^) together with a broad and very intense band at about 3360 cm^–1^.

The band profiles analysis performed in the literature reveals that the weak band is composed of two components assigned to monomers (3624 cm^–1^) and end chain (proton acceptor of hydroxyl terminal) of linear dimers, respectively.^9,10^ The strong band has been described in terms of three components related to linear dimers and cyclic tetramers and hexamers (respectively, at 3520, 3410, and 3290 cm^–1^). These values are very close by both techniques.^9−11^

It appears that Raman spectroscopy is more sensitive to the presence of monomers and small aggregates, whereas associated species mainly influence the infrared spectra (Figure 1a).^65,66^ This finding is particularly supported by considering the domain of the γ_OH_ out-of-plane vibration. In infrared, this vibration has been found at about 250 cm^–1^ for TBH monomers in low-temperature matrices (10 K) and at about 640 cm^–1^ for TBH diluted in carbon tetrachloride.^1^ In the neat liquid, the broad band observed at about 635 cm^–1^ in infrared spectroscopy is clearly the spectral signature of hydrogen-bonded associated molecules (Figure 1b). Incidentally, we note that this band can be nicely fitted by a single Gaussian profile having an full width half height (FWHH) of about 170 cm^–1^. This result which differs from the composite profile of ν_OH_ stretching vibration suggests that the line-shape is inhomogeneously broadened and reflects a rather static distribution (on the timescale of the experiment) of out-of-plane vibrations. In marked contrast, this broad band is absent in Raman spectroscopy,^2^ presumably due to its low activity (Figure 1b, see Section 4). This result nicely illustrates both the difference and the complementarity between the spectroscopic techniques, as previously illustrated for the ν_OH_ stretching vibration.

#### 3.1.2 TBF

**Figure 2:**
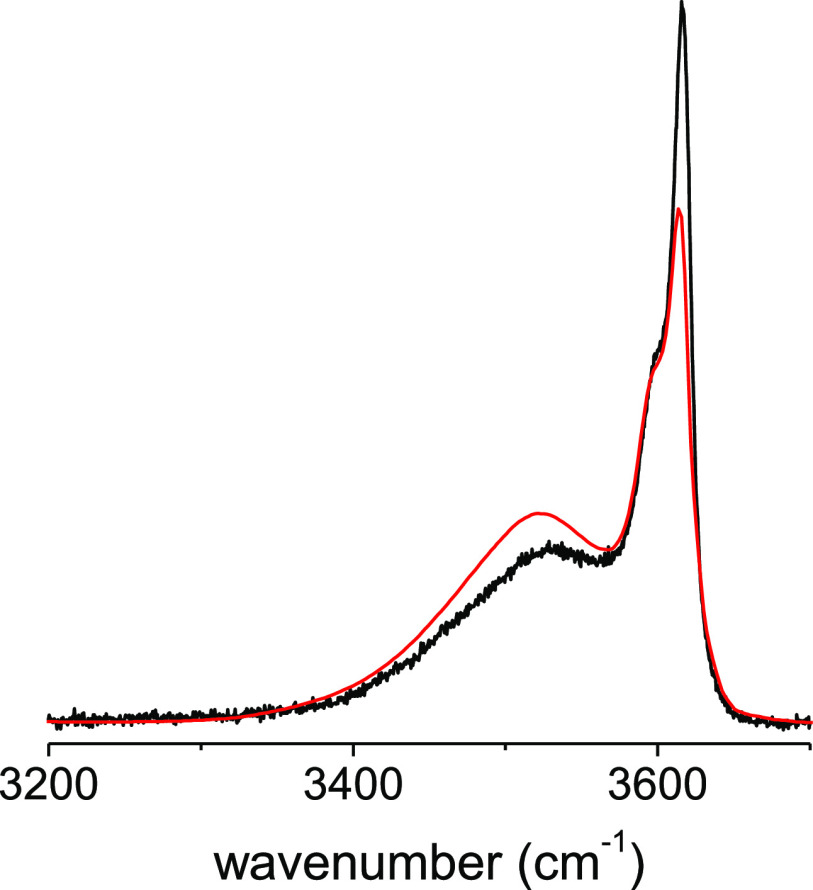
Raman (black) and infrared (red) spectra of pure TBF in the spectral domain of ν_OH_ stretching vibration.

The Raman and infrared spectra of the ν_OH_ stretching vibration of liquid TBF are displayed in Figure 2. The two spectra have very similar shape and exhibit a narrow component having a doublet-like structure, accompanied by a broad component at lower wavenumbers. The higher-frequency component was assigned to monomers (3615 cm^–1^) and its shoulder to the non-H-bonded end groups of linear H-bond chains, the so-called “open end OH” (3605 cm^–1^). The broad feature at about 3524 cm^–1^ was assigned to higher oligomers.^47,52^

It was recently shown on the basis of a multitechnique spectroscopic approach, analyzed from DFT calculations and molecular dynamics, that TBF consists mainly of monomers having a population greater than 75%, whereas the main contribution of oligomers comes essentially from dimers. The main conclusion was that TBF is a poorly associated alcohol.^47,52,55^ It is on this unusual result, at variance with the fact that the liquid phase of usual hydrogenated alcohols is characterized by a large network formed by hydrogen-bonded molecules, that the strategy of this work is based.

For TBF in the gaseous phase, the γ_OH_ vibration (out-of-plane vibration) and the δ_OH_ bending (in-plane vibration) were observed in infrared spectroscopy at 252 and 1382 cm^–1^, respectively.^47^ If these vibrations were affected by the aggregation in liquid phase, as it happens in TBH,^1,55^ they would appear in the spectral domain 600–800 cm^–1^ and shifted toward a higher wavenumber by about 70 cm^–1^, respectively.^1^ While the δ_OH_ bending is observed at about the same frequency (1380 cm^–1^), the γ_OH_ vibration was not observed, either because it is not affected by hydrogen bonding or its activity is too low to be detected.^55^ These results, showing that molecular association in TBF is very weak, further support the conclusion reached from the study of the ν_OH_ stretching vibration that TBF is a poorly associated alcohol.

### 3.2 TBH–TBF Mixtures

#### 3.2.1 Raman Spectroscopy

**Figure 3:**
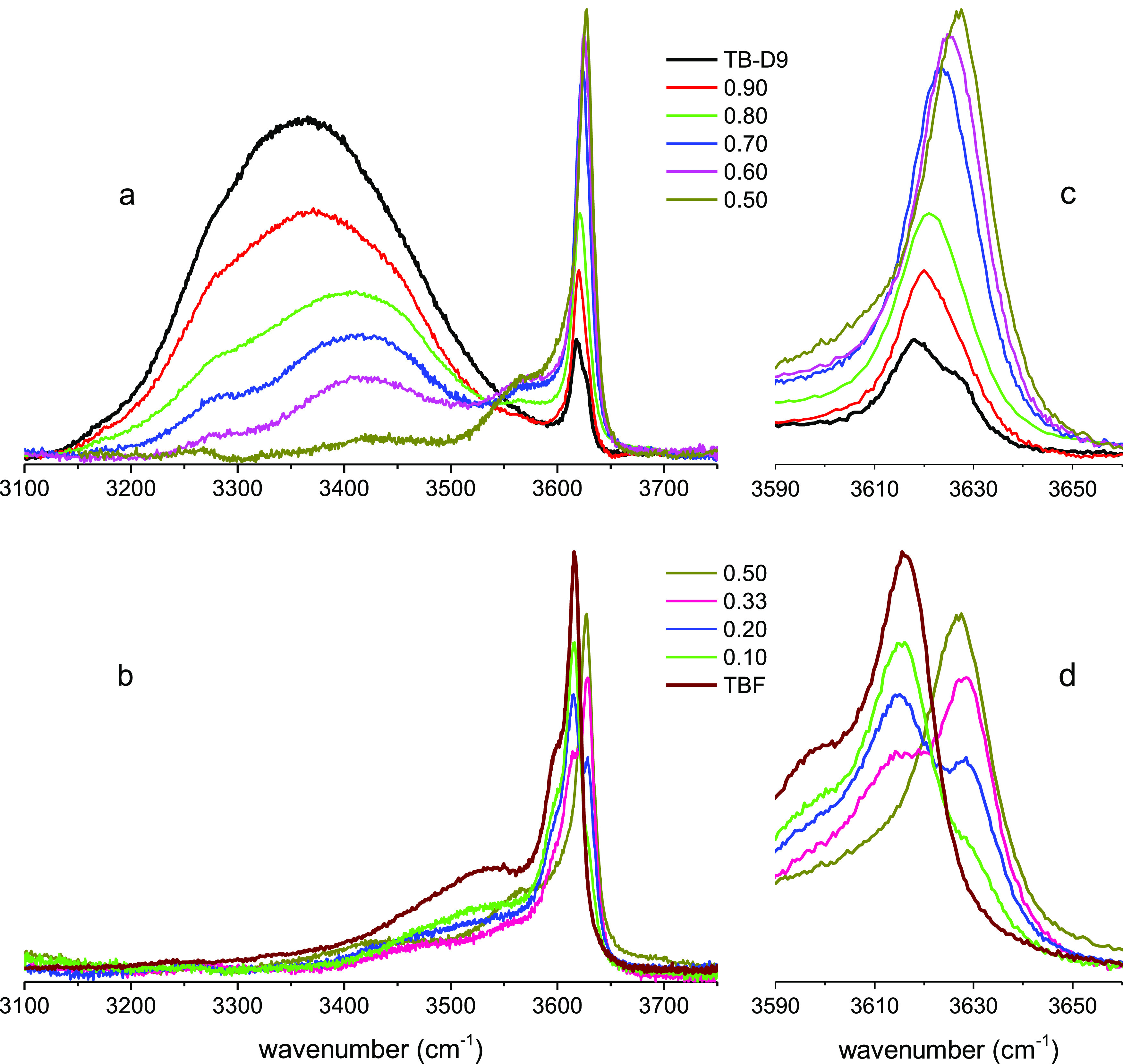
Raman spectra of the ν_OH_ stretching band of liquid mixtures of TB-D9 in TBF: (a) (molar fraction x_TB-D9_: 1; 0.90; 0.80; 0.70; 0.60; 0.50) and (b) (x_TB-D9_: 0.50; 0.33; 0.20; 0.10, 0). The spectral domain centered at about 3620 cm^–1^ is displayed in a magnified scale (c, d).

The Raman OH-stretching bands of mixtures of liquid *tert*-butanol (TB-D9) with increasing amounts of TBF are displayed in Figure 3a,b, using as a reference the intensity of the spectra of the C–D_3_ stretching vibration. This standard procedure is based on the hypothesis that the activity of these modes is unaffected by the dilution.^9,10,12,13^

Sizeable variations of the spectral shapes and intensity of the spectra are observed. The intensity of the broad composite profile centered at about 3360 cm^–1^ in pure *tert*-butanol and assigned to cyclic hexamers (3290 cm^–1^) and tetramers (3410 cm^–1^) strongly decreases and continuously shifts toward a higher frequency to almost vanish at the equimolar concentration (Figure 3a). Increasing again the concentration in TBF (0.5–0.1 molar fraction in TB-D9) shows that it is only in the spectral region *ca.* 3450–3650 cm^–1^ that two features are observed (Figure 3b). The spectrum is constituted by a faint band at about 3550 cm^–1^ accompanied by a narrow composite intense feature at about 3630 cm^–1^. The overall spectral shape appears progressively similar to the pure TBF spectrum with increasing TBF concentration. The details of the concentration evolution of the composite intense feature are better appreciated in Figure 3c,d. This feature is made of two components in pure *tert*-butanol, which progressively collapse leading to a single line at an equimolar concentration (Figure 3c). For more diluted solution, a splitting of this single line is observed, giving rise to two components. The component at about 3630 cm^–1^ will progressively vanish and is barely observable at 0.10 molar fraction (Figure 3d). The other component at about 3615 cm^–1^ has an increasing intensity and approaches the spectrum observed for pure TBF (Figure 3d).

Several conclusions can be obtained from this study. First of all, the continuous dilution of *tert*-butanol by TBF leads to the destruction of higher-order oligomers. The concentration of hexamers and tetramers oligomers vanishes progressively and is almost negligible at the equimolar concentration. Concomitantly, the population of small oligomers, namely, linear dimers and monomers increases as expected.

**Figure 4:**
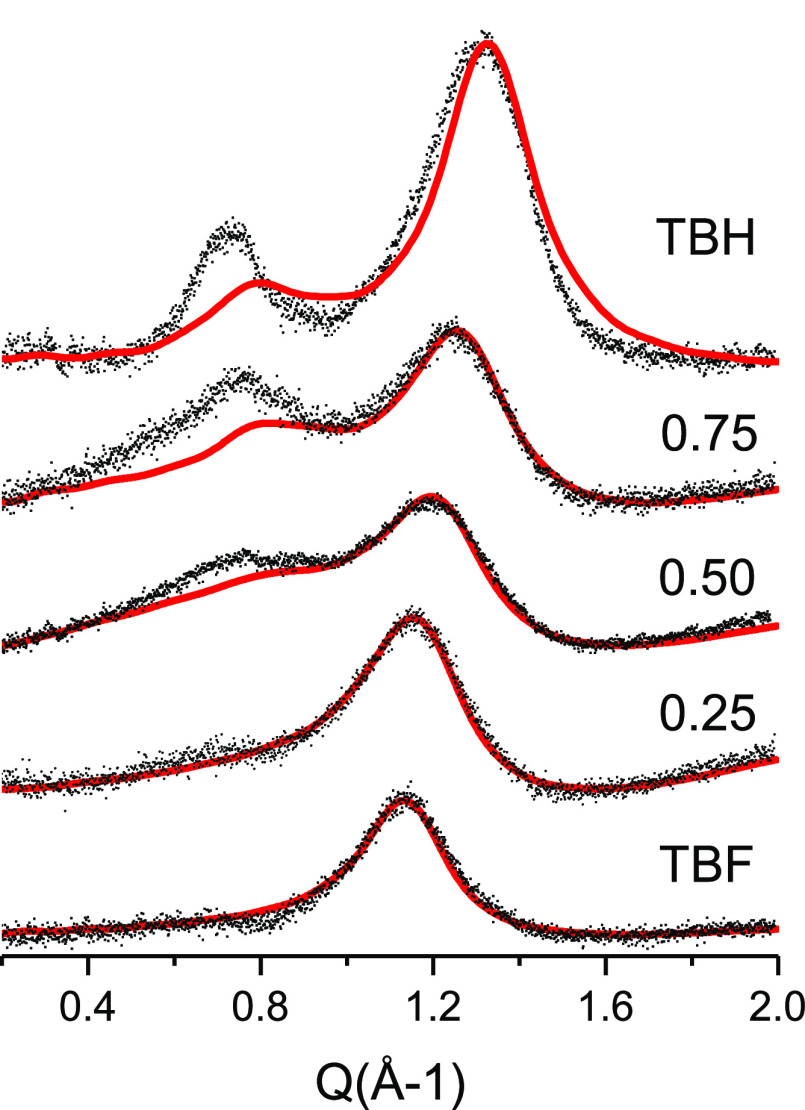
Experimental (black dots) and calculated (red line) X-ray diffraction patterns of liquid TBH–TBF mixtures: *x*_TBH_: 1; 0.75; 0.50; 0.25; 0.

To confirm the disappearance of large oligomers, we have performed X-ray diffraction of the pure alcohols and their mixtures (Figure 4). For pure liquid TBH, we found a main diffraction peak at momentum transfer *Q* of about 1.3 Å^–1^, accompanied by a pre-peak at about 0.7 Å^–1^. Structural studies show that liquid TBH forms supramolecular clusters organized in cyclic small reverse micelle-like aggregates of four to six molecules having all their hydrogen-bonding sites together, with the methyl groups pointing to the outside.^13,19,27,28^ The values of the diffraction peaks are consistent with those reported by Morineau et al.^13^ for the static structure factor obtained by neutron diffraction. The pre-peak was interpreted as due to the existence of an intermediate range ordering between adjacent cyclic clusters. The main peak results from both intermolecular and intramolecular contributions.

Upon dilution in TBF, we observed a decreasing intensity of the two peaks and a slight shift of the position of the main diffraction peak showing that intermolecular correlations are progressively disappearing. This observation is consistent with the solvent effect previously reported.^13^ Of importance is to notice that the intensity of the pre-peak almost vanishes at the equimolar concentration and is not detectable at higher dilution. In particular, for diluted solutions (*x*_TBH_ = 0.25), the X-ray pattern is very similar to that of pure TBF.

The absence of the pre-peak has been taken from simulation studies as an indication that the local organization coming from hydrogen-bonding molecules has disappeared. It was inferred that the whole atomic distribution was as random as for the nonhydrogen-bonding sites.^27,28^ However, this conclusion is at variance with the Raman analysis of the ν_OH_ stretching, which provides direct insight into H-bond formation. Indeed, at dilutions higher than equimolar, Raman results show that both dimers and monomers should be present in the solution.

In conclusion, Raman results combined with diffraction measurements show that the population of large TBH oligomers is vanishing with the dilution in TBF. However, this approach puts the emphasis on the existence of TBH aggregates as if they were observed in noninteracting “matrix” or solvent, neglecting the interactions between TBH and TBF molecules. Nevertheless, hetero-oligomers must be present because TBF is not a noninteracting solvent.

To clarify this issue, we have used infrared spectroscopy, which is more sensitive to complex formation than Raman spectroscopy.

#### 3.2.2 Infrared Spectroscopy

##### 3.2.2.1 ν_OH_ Stretching Spectral Domain

**Figure 5:**
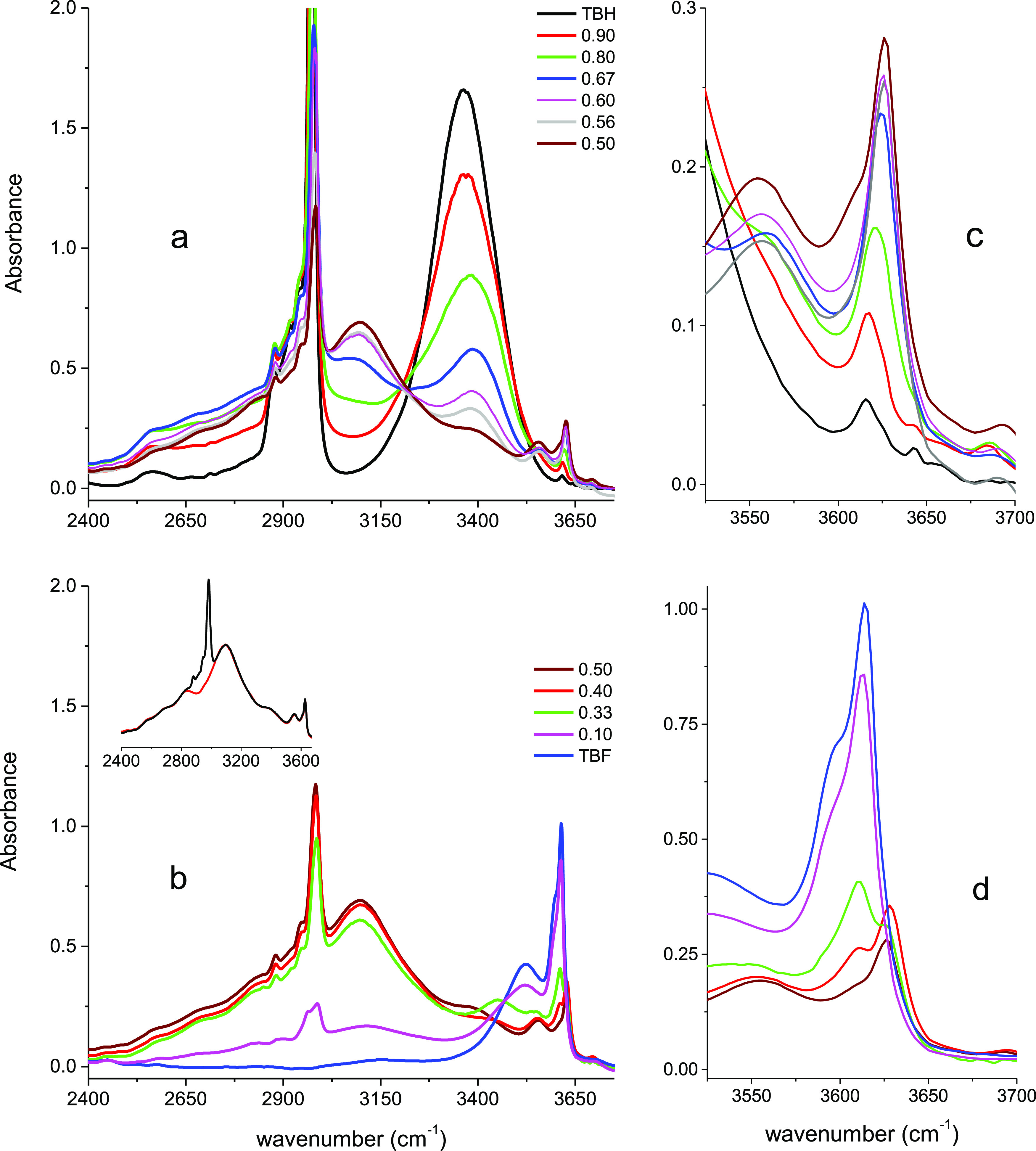
Infrared spectra in the region of the ν_OH_ stretching band of liquid mixtures of TBH in TBF: (a) (*x*_TBH_: 1; 0.90; 0.80; 0.67; 0.60; 0.56; 0.50) and (b) (*x*_TBH_: 0.50; 0.40; 0.33; 0.10; 0). The spectral domain centered at about 3620 cm^–1^ is displayed in a magnified scale (c, d). Equimolar TBH–TBF (black) and TB-D9-TBF (red) mixtures are compared in the inset of (b).

The infrared spectra of the ν_OH_ stretching band of TBH measured by adding increasing amounts of TBF are displayed in Figure 5a,b. The variations of the shapes and intensities of the spectra observed in the range 3250–3650 cm^–1^ are rather similar to those previously described for Raman spectroscopy.

The intense and broad component observed in pure TBH at about 3360 cm^–1^, assigned to cyclic tetramers and hexamers, strongly decreases and continuously shifts toward higher frequency to almost vanish at an equimolar concentration (Figure 5a,b) as it was observed in Raman.

Similarly, the features observed in the spectral domain 3550–3700 cm^–1^ (Figure 5c,d), also present the same trend in shape and intensity as those reported for Raman spectroscopy. They also reflect the same complex evolution and competition between partitioning of OH end chain and small oligomers (monomers and dimers).

However, a new and very broad feature ca. 3100 cm^–1^ is detected in infrared, but not in Raman. This feature is observed from very low dilution of TBH by TBF (*x*_TBH_ = 0.9) with an intensity strongly increasing up to equimolar concentration. Concomitantly, the intensity of the band assigned to cyclic tetramers and hexamers decreases and vanishes at the equimolar concentration (Figure 5a). Beyond this value, the intensity of the band centered at 3100 cm^–1^ remains almost independent of the concentration up to *x*_TBH_ = 0.33 and then decreases to vanish in pure TBF (Figure 5b).

Prior to the interpretation of our results, it is worth pointing out a number of facts. Raman and infrared spectroscopy allow us to conclude in a consistent manner that larger clusters (mostly tetramers at room temperature) of TBH are destroyed upon dilution by TBF. The evolution of the spectra shows that it is at the equimolar concentration that small oligomers, monomers and dimers, become predominant. The disappearance of intercluster correlation of large TBH aggregates observed by X-ray diffraction reinforces this view of the solvation processes in the mixture. These findings are well known in the case of speciation of alcohol upon dilution in a “non-” or weakly interacting solvent as, for instance, TBH molecules diluted in dimethylbutane^11,12^ and in methylcyclohexane or toluene.^13^

**Figure 6:**
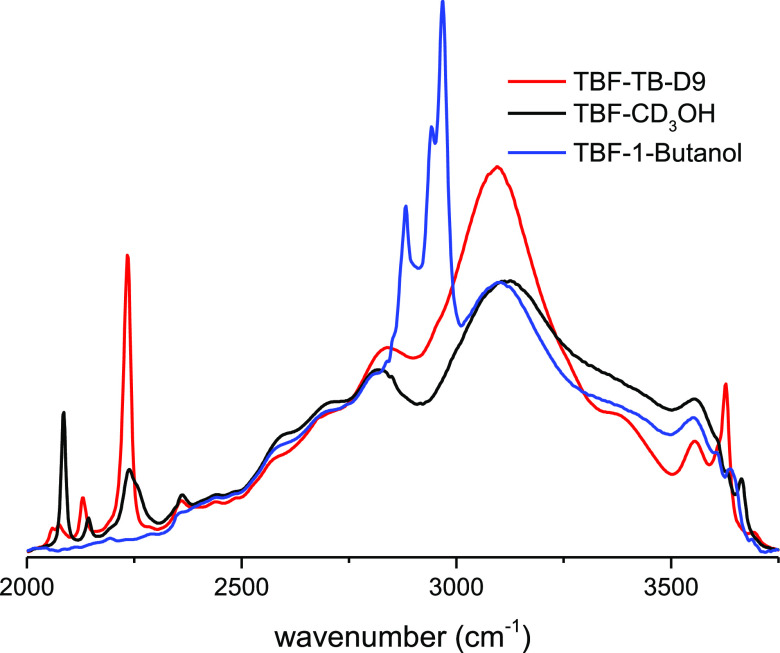
Infrared spectra of equimolar mixtures of TBF–CD_3_OH (black) and TBF-1-butanol (blue) compared with that of TBF–TBH (red).

However, the existence of the new intense and broad ν_OH_ stretching band at 3100 cm^–1^ needs to be understood to extend the previous view of the speciation. For this purpose, we have also studied equimolar mixtures of TBF with methanol (CD_3_OH) and with 1-butanol by infrared spectroscopy. We found also a new intense and broad feature with a similar composite band shape in the same spectral domain in all of the mixtures (Figure 6).

This new broad band is assigned to the ν_OH_ stretching band of the TBF molecule in interaction with hydrogenated alcohol molecules. It presents the well-known signatures characteristic of hydrogen bonding, namely, a large shift toward lower frequencies, a strong integrated intensity, and a very large full width (typically 460 cm^–1^).^67−81^

**Figure 7:**
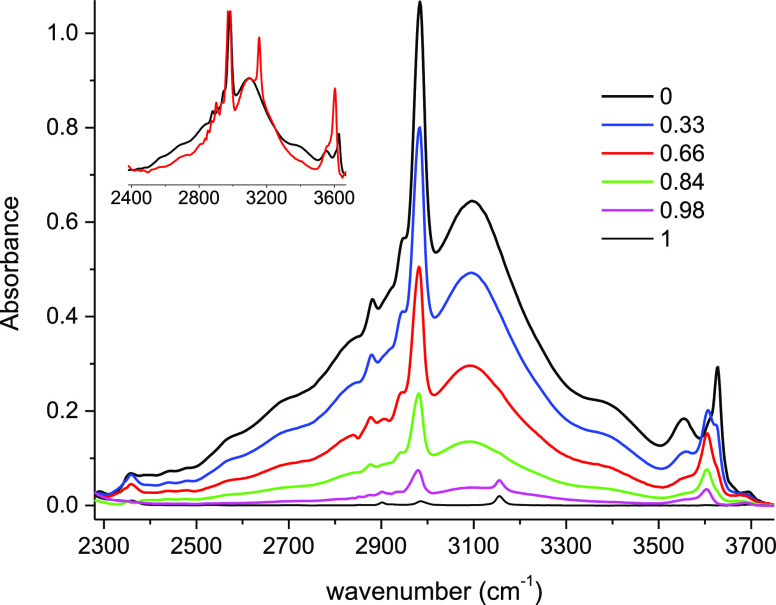
Infrared spectra of TBH–TBF equimolar mixtures diluted in CDCl_3_ as a function of the CDCl_3_ concentration (*x*_CDCl_3__: 0, 0.33; 0.66; 0.84; 0.98; 1). The scaled spectrum of the more diluted mixture (*x*_CDCl_3__ = 0.98, red) is compared with that of the TBH–TBF equimolar binary (black) in the inset.

To get additional insight into the origin of this band, we have proceeded to the measurement of the infrared spectra of TBH–TBF equimolar binary mixtures diluted in a polar solvent (CDCl_3_). We observed that the shape of the composite profile is not affected even at high dilution *x*_CDCl_3__ = 0.98 (Figure 7, inset). Using these experiments, we have plotted the variation of the integrated intensity of the broad feature versus the TBH molar concentration, and a good linear trend is observed (Figure S2).

In a second series of experiments, we have proceeded by diluting the TBH–TBF binary mixtures in CDCl_3_ but with an excess of one of the two alcohols. Again, the infrared spectra display the broad feature at 3100 cm^–1^ with the same line-shape as observed in equimolar binary mixtures but accompanied by a spectral signature characteristic of the alcohol in excess (Figure S3).

The fact that the band shape is almost unaffected by the dilution, together with the observation that its intensity evolves in a linear manner over a large range of concentrations of the polar solvent, suggests a rather robust molecular distribution to sustain the dilution with an interacting polar solvent.

## 4 Simulation Results

**Figure 8:**
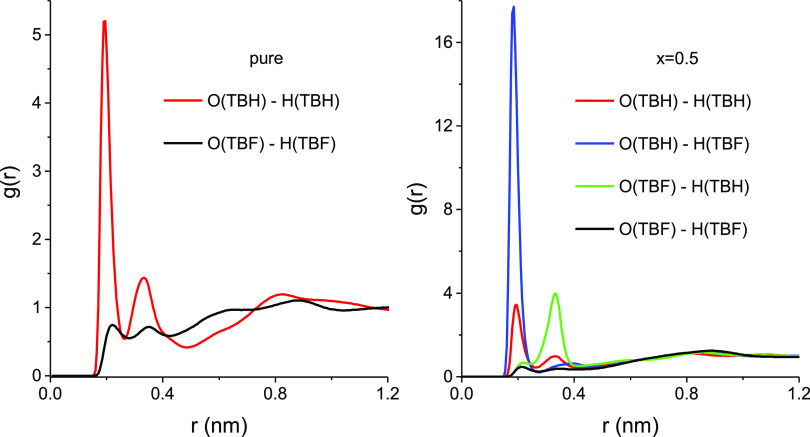
Intermolecular radial distribution functions g_O···H_ between the oxygen and hydroxyl hydrogen atoms for the equimolar TBH–TBF mixture (right) compared with those for the pure liquids (left).

The structure of the TBH–TBF liquid mixtures was further studied by atomistic molecular dynamics simulations. Partial intermolecular radial distribution functions (rdf or *g*(*r*)) were obtained from the simulated trajectories, providing molecular insight into the local liquid structure. Figure 8 shows the rdf between oxygen and hydroxyl hydrogen atoms, both in the pure compounds (left) and in the equimolar mixture (right). As observed in our previous work,^55^ the intense first peak of the O–H rdf in pure TBH indicates that the structure of the hydrogenated alcohol is dominated by the presence of hydrogen bonds, whereas its fluorinated counterpart is essentially monomeric, as seen in its O–H rdf, which is lower than 1 up to a long distance, commensurate with the molecular diameter. When the two alcohols are mixed, a total of four types of hydrogen bonds may form, corresponding to all of the possibilities of both alcohols acting as hydrogen-bond donors and acceptors. The rdfs for these combinations in the equimolar mixture are represented in Figure 8 (right), where it can be seen that the distribution of hydrogen bonds (which can be identified with the integral of the first rdf peak) is hugely asymmetric. The dominant feature is now the rdf peak that corresponds to the hydrogen bond between the hydrogen of TBF and the oxygen of TBH, followed by the much less intense rdf of the hydrogen bond between two TBH molecules. The first peaks of the two rdfs, where TBF acts as a hydrogen-bond acceptor, are very weak. In these mixtures, TBF confirms its very low hydrogen-bond acceptor capability but reveals an intense donor ability toward the hydrogenated alcohol.

**Figure 9:**
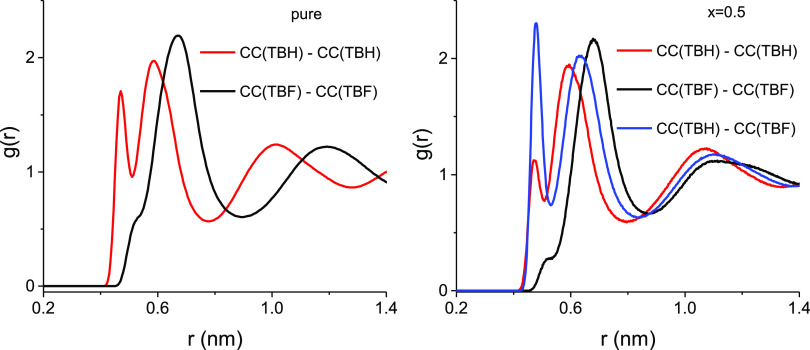
Intermolecular radial distribution functions g_CC···CC_ between the central carbons (CC) for the equimolar TBH–TBF mixture (right) compared with those for the pure liquids (left).

The rdf between the central carbon (CC) atoms of TBH and TBF in the equimolar mixture, in comparison with those obtained for the pure compounds, are shown in Figure 9. As seen previously,^55^ the CC rdf in pure TBH displays two very well-defined peaks at short distances, corresponding to the two distinct ways in which these molecules interact. The first of these peaks may be identified with the CC–CC distance between two hydrogen-bonded molecules and the second to the different “back-to-back” configurations when two TBH molecules contact through the methyl groups. On the contrary, this rdf in TBF has only a small shoulder after the lift-off, identified with hydrogen-bonded contacts, followed by a large peak that resembles the rdf of a simple spherical fluid. In the equimolar mixture, these two “homo” rdfs decrease, especially the first peak of the TBH–TBH pair and the shoulder of the TBF–TBF peak, whereas on the other hand, the new “hetero” rdf between the two different compounds is very intense and structured. These results show that the short-range organization of the mixture is achieved by the destruction of the structure of pure TBH and newly determined by the formation of new “hetero” hydrogen bonds.

The X-ray diffraction spectra of the studied solutions were also predicted by the simulation results. These spectra were calculated from the molecular dynamics trajectories using the TRAVIS software, for large systems with a total of 3000 molecules. As can be seen from Figure 4, the calculated spectra remarkably reproduce the experimental results. Both the position of the peaks and their evolution with the composition of the system are completely and quantitatively predicted, validating the molecular models and the structural interpretations obtained from the MD simulations.

The liquid structure of the simulated systems was further characterized by describing the distribution of sizes, composition, and topologies of the hydrogen-bonded aggregates, which were also identified in the spectroscopic study. This analysis was done with an in-house built program, which scans the stored configurations and searches for aggregates considering that two molecules are neighbors and belong to the same cluster if the hydroxyl hydrogen of one of them is closer than 0.27 nm to the oxygen atom of the other, and outputs histograms of aggregate sizes, aggregate compositions (number of TBH and TBF molecules) for each size, and aggregate topologies (linear, cyclical, etc.) for each size.

**Figure 10:**
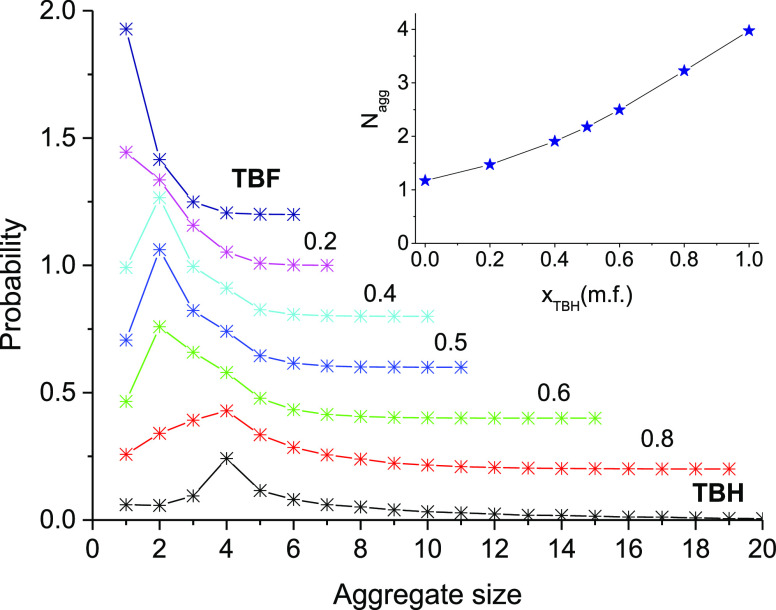
Probability of finding a molecule in a hydrogen-bonded aggregate of size *n*, for several TBH–TBF compositions, determined from the MD trajectories. The data sets were displaced vertically for clarity. Average aggregation number *N*_agg_ as a function of composition in the inset.

The inset of Figure 10 shows the average aggregation number *N*_agg_ as a function of mixture composition. It can be seen that this number decreases in a monotonous manner from pure TBH (where *N*_agg_ = 3.97) to pure TBF, where almost all molecules are found as monomers (*N*_agg_ = 1.17). The full distribution of aggregate sizes for the studied mixture compositions is seen in the main part of Figure 10, where the probability *p*(*n*) of finding a molecule in an aggregate of size *n* is plotted as a function of *n* (the curves have been displaced vertically for legibility). This probability is defined as
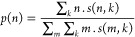
where *s*(*n*,*k*) represents the number of aggregates of size *n* in the configuration *k*. The full distribution of aggregate types, by size, composition, and topology is given in Table S2.

As shown in our previous work, the two neat alcohols studied show very different aggregation behaviors.^55^ Although TBH tends to form large clusters with a broad distribution of sizes and almost 80% of the molecules belong to tetramers or larger aggregates, pure TBF appears essentially in the monomeric form (73% of the molecules), with small proportions in dimers (22%) and trimers (5%) and a residual population in larger aggregates.

In the mixtures, when the proportion of TBF increases, the size distribution is displaced toward lower aggregation numbers and the larger clusters progressively disappear. When 20% of TBH is replaced by TBF (*x*_TBH_ = 0.8), the percentage of monomers remains essentially constant (6%), but the small clusters, dimers (14%), and trimers (19%) significantly increase in importance. For this composition, tetramers remain the most important aggregate size, accounting for 23% of the molecules, but the number of larger aggregates greatly decreases, with now only 4% of the molecules clustering in aggregates of size 10 or larger (*vs* 20% in pure TBH) and, while cyclic aggregates still appear, the linear configuration is now the dominant topology for all sizes. Looking at the composition of the aggregates, the most probable clusters in this mixture are the 2:1 (TBH:TBF) trimer and the 3:1 tetramer, each accounting for around 14% of the mixture, and more than 10% of the molecules aggregate as heterodimers.

If the TBF proportion is further increased (*x*_TBH_ = 0.6, 0.5, and 0.4), the aggregation behavior significantly changes. As can be clearly seen in Figure 10, the preferred cluster size is now the dimer, with a population that increases to 36% for *x*_TBH_ = 0.6, 46% for *x*_TBH_ = 0.5, and 47% for *x*_TBH_ = 0.4. Nearly all of the dimers (more than 95%) are composed of one molecule of TBH and one molecule of TBF, making the heterodimer (F–H) the dominant type of aggregate for these compositions. The proportion of larger clusters progressively decreases with the addition of TBF, but trimers (2:1 and 1:2) and tetramers (especially 2:2 and 3:1) remain significant in this concentration range. The proportion of monomers in the mixture also increases with the concentration of the fluorinated alcohol (7, 11, and 19% for *x*_TBH_ = 0.6, 0.5, and 0.4, respectively).

**Figure 11:**
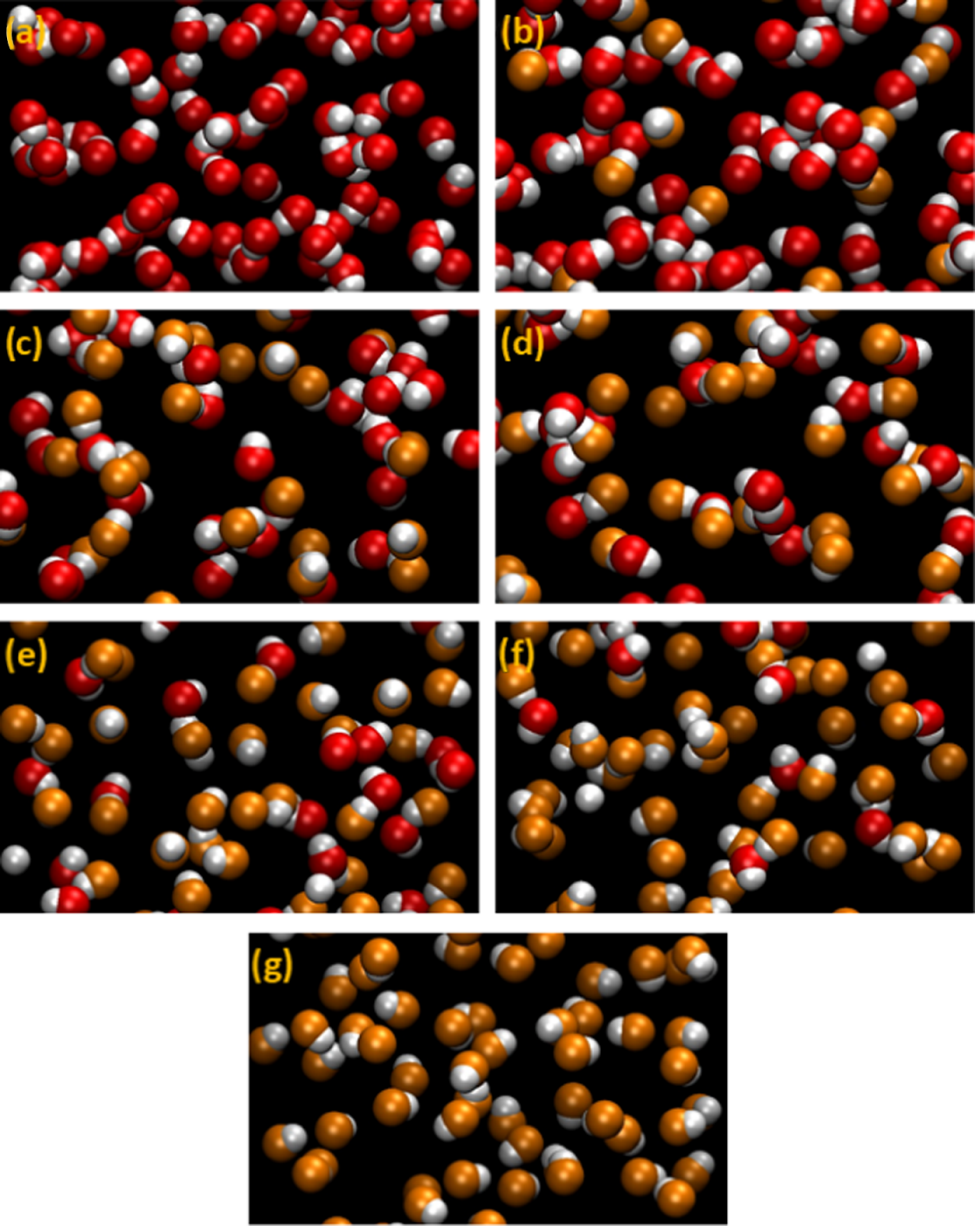
Simulation snapshots of the studied systems. For the sake of clarity, only the hydroxyl groups are represented as van der Waals spheres, with hydrogen atoms in white, the oxygen atom of TBH in red, and the oxygen atom of TBF in orange. Approximately equal volumes of each system are represented: (a) pure TBH, (b) *x*_TBH_ = 0.8, (c) *x*_TBH_ = 0.6, (d) *x*_TBH_ = 0.5, (e) *x*_TBH_ = 0.4, (f) *x*_TBH_ = 0.2, and (g) pure TBF.

The *x*_TBH_ = 0.2 mixture displays a third type of behavior, approaching that of pure TBF, where the most frequent species is the monomer and clusters larger than tetramers are almost nonexistent. In this mixture, the most probable aggregated form is still the heterodimer, accounting for 25% of the total molecules, but the most frequent species in the mixture is already the TBF monomer. This evolution in the aggregation behavior for the different compositions is illustrated in Figure 11, where it can be seen that the large aggregates observed in pure TBH (panel a) progressively disappear with the addition of TBF, being replaced by an increasing number of dimers and monomers. The tendency of TBF to act essentially as a donor of hydrogen bonds is very evident in all mixtures, as well as the abundance of the F–H heterodimer, especially in the intermediate range of compositions.

**Figure 12:**
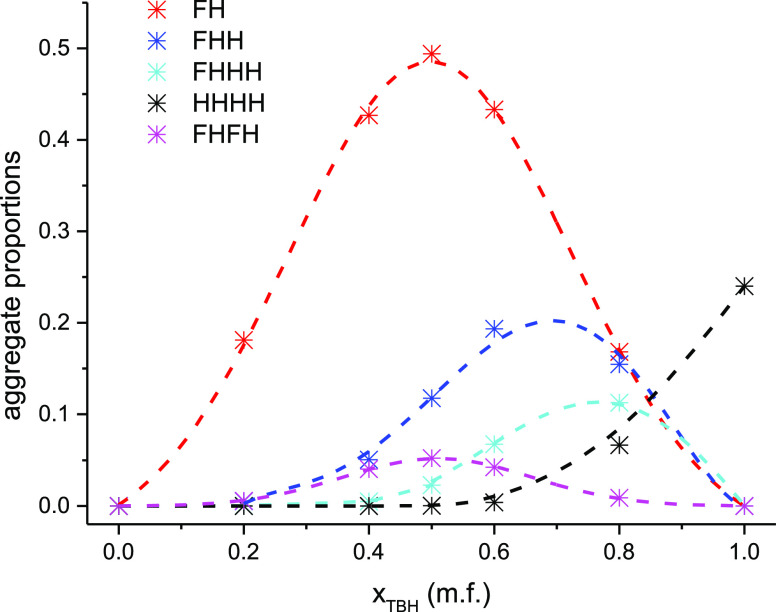
Evolution with the concentration of the probability of finding the most relevant aggregates.

The evolution of the population of the most relevant aggregates with the composition of the system is given in Figure 12 (Table S2). The heterodimer is the most probable aggregated form in all of the mixtures and is the only significant aggregate at low TBH compositions. Interestingly, its probability curve displays a Gaussian shape centered at *x* = 0.5. The proportions of higher aggregates increase with the TBH concentration, and their relative importance varies with the system composition.

This analysis can be further interpreted by considering the very asymmetric hydrogen bonding character of TBF, as it is a very strong hydrogen-bond donor due to the electron-withdrawing character of the CF_3_ groups and higher acidity of the hydroxyl proton, but a weak acceptor due to the smaller partial charge of the oxygen atom and to its high stereochemical impediment. In the mixtures, the TBF molecules tend to act solely as hydrogen-bond donors, being essentially found at the “beginning” of the hydrogen-bond chains and thus hindering the formation of large clusters. The very high stability of the TBF (donor)–TBH (acceptor) heterodimer, which is supported by the quantum chemical calculations, makes this species the dominant form of aggregation in an extended range of concentration (0.3–0.7 m.f.) centered in the vicinity of the equimolar fraction.

## 5 Discussion

We are now able to interpret the infrared spectroscopic results which demand the knowledge of both the population and spectral activity of the different aggregates existing in the mixtures. As seen before, the MD calculations are able to provide a detailed picture of the speciation in the mixtures. To access the spectral activities, we have resorted to a DFT approach.

First of all, the binding energy of the linear and cyclic TBH *n*-mers (*n* = 1–6), TBF *n*-mers (*n* = 1–3), and all of the possible hetero-oligomers (n = 2–6) was calculated. The details of these calculations are presented in the SI. First, the calculations show that the heterodimer in which the TBF molecule is the H-bond donor (noted here as FH) has a binding energy twice that of the homodimers of TBH (HH) and TBF (FF). Next, for each type of aggregate, we have selected the most stable ones. It emerges that the relevant species are the linear dimer (FH), the linear and cyclic trimers (FHH, FFH), and the linear and cyclic tetramers (FHFH). The calculated frequency and activity of the ν_OH_ and γ_OH_ vibrations in the oligomers are gathered in Tables S3–S6.

**Figure 13:**
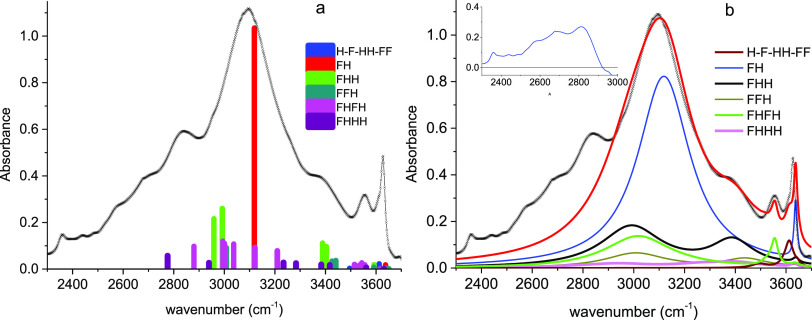
Infrared band of the TB-D9 – TBF equimolar mixture in the spectral domain 2400–3700 cm^–1^: (a) Stick diagram presenting the contribution of relevant aggregates calculated from their vibrational frequencies and activity (DFT) and their probability (MD); (b) Band-shape analysis adjusting the broadening of Lorentzian profiles. The difference between the experimental profile (black, −x−) and the sum of all of the displayed contributions (red) is presented in the inset. H-F-HH-FF refers to the sum of contributions of TBH monomers, TBF monomers, HH homodimers, and FF homodimers, respectively.

The analysis of the new broad band detected in the equimolar mixture was performed using the frequency and activity calculated by DFT and the relative population of the relevant aggregates obtained by MD. The resulting calculated profile displayed using a stick representation for each type of aggregate is compared with the experimental profile (Figure 13a). Clearly, the dominant contribution comes from the FH heterodimer. The contributions of the other species appear flanking each side of the main line with a much lower intensity. We have then described the experimental profile by fitting Lorentzian components for which only the broadening was adjusted. It appears that to achieve a good fit (Figure 13b, Table S7), five components are needed, corresponding to monomers and associated species ranging up to tetramers. This last representation is consistent with the stick diagram and leads to an integrated view of the spectral contributions of the different types of aggregates in the overall profile. Furthermore, the existence of a weak composite substructure appearing on the lower-frequency side of the profile can be better appreciated using this representation compared to a stick diagram. We note that this substructure is also observed in other equimolar mixtures (TBF-methanol, TBF-1-butanol, Figure 6).

The main component is associated with the heterodimer FH and contributes to about 50% percent of the total intensity. The linear trimers FHH and the linear tetramers FHFH are the most relevant oligomers with contributions of about 20 and 10% of the total intensity, respectively. The profiles associated with the vibrators of the oligomers in which the TBF molecule is the H-bond donor are found to be very large (width of 260–290 cm^–1^). In contrast, for the TBH molecule, engaged in the oligomers as H-bond acceptor, the widths are narrower (30–70 cm^–1^, Table S7). The difference between the experimental profile and the sum of the contributions considered is presented in the inset of Figure 13. The decomposition of the composite substructure observed at about 2500–2900 cm^–1^ involves narrower components (widths 90–150 cm^–1^) than those associated with the H-bond donor oligomers. In this spectral domain, higher-order oligomers could contribute, but their population is almost negligible. Even if they were relevant, the profiles associated should be very broad. Therefore, we can rule out any significant contribution of higher-order oligomers.

Concerning the substructure, we may invoke that it originates from both overtones of δ_COH_ modes of TBF in a heterodimer and combination bands of this mode either with the ν_CO_ stretching mode or with δ_CH_3__ modes of the interacting proton acceptor TBH (Table S6). However, we emphasize that the interpretation of such substructure would require a dedicated study, which is beyond the scope of this work.

## 6 Conclusions

We may conclude that the infrared spectra measured for the liquid TBH–TBF mixtures possibly result from the superimposition of the spectra of small hydrogen-bonded oligomers. Concerning the speciation of the mixture, from MD simulations, we can infer that in a rather extended range of concentration, centered in the vicinity of the equimolar one, say from 0.3 to 0.7 m.f., the liquid mixture is mostly composed of heterodimers, TBF monomers, TBH monomers, and dimers. Of course, other species like small heteroclusters, as well as TBF monomers, TBH monomers, and dimers are present but in small proportion. For TBH concentrations lower than 0.3 m.f., TBF monomers are predominant, whereas for concentrations higher than 0.7 m.f., the state of aggregation of the mixture should progressively approach that of the pure TBH.

This work shows the interest in using perfluoro *tert*-butanol (TBF) in the studies of the state of aggregation of liquid hydrogenated alcohols (TBH, methanol, and 1-butanol).

In “inert” solvents, the breaking of the H-bond network only leads to the formation of monomeric species at high dilution. In contrast, for TBF, due to its small p*K*_a_ value, in the presence of a hydrogenated alcohol (generally more basic), the formation of heterodimers is predominant and persists even at high dilution in polar solvents. This alcohol offers a unique way to study aggregation in H-bonded systems, from liquid to gaseous phases, and merits to be further carefully considered for the possibilities offered in both theoretical and experimental investigations.

## Supporting Information Available

Additional experimental details and results, OPLS-AA functions and parameters, probability of finding hydrogen-bonded aggregates as a function of the mixture composition, details and results of DFT calculations, and details of the IR broad-band-shape analysis (PDF)
