# Supplementary material for: Breaking the Structure of Liquid Hydrogenated Alcohols Using Perfluorinated tert-Butanol: A Multitechnique Approach (Infrared, Raman, and X-ray Scattering) Analyzed by DFT and Molecular Dynamics Calculations
Source: J Phys Chem B. 2022 Mar 1;126(9):1992–2004. doi: 10.1021/acs.jpcb.1c10776 (PMC9776561; doi:10.1021/acs.jpcb.1c10776)
Supplement: Supplementary file 1 — jp1c10776_si_001.pdf [file jp1c10776_si_001.pdf]

SUPPORTING INFORMATION

Breaking the Structure of Liquid Hydrogenated Alcohols Using Perfluorinated Tert-butanol: A Multi-technique Approach (Infrared, Raman and X-Ray Scattering) Analysed by DFT and Molecular Dynamics Calculations

M. Isabel Cabaço[a,b*], Marcel Besnard[a,c], Carlos Cruz[a], Pedro Morgado[d], Gonçalo M. C. Silva[d], Eduardo J. M. Filipe[d], João A. P. Coutinho[e], Yann Danten[c]

[a]CeFEMA, Centro de Física e Engenharia de Materiais Avançados, Departamento de Física, Instituto Superior Técnico, Universidade de Lisboa, 1049-001 Lisboa, Portugal
[b]Laboratory of Instrumentation, Biomedical Engineering and Radiation Physics (LIBPhys-UNL), Department of Physics, NOVA School of Science and Technology, NOVA University Lisbon, 2829-516 Caparica, Portugal
[c]GSM Institut des Sciences Moléculaires, CNRS (UMR 5255), Université Bordeaux I, 351, Cours de la Libération 33405 Talence Cedex, France
[d]Centro de Química Estrutural, Instituto Superior Técnico, Universidade de Lisboa, Av. Rovisco Pais 1, 1049-001 Lisboa, Portugal
[e]CICECO, Departamento de Química, Universidade de Aveiro 3810-193 Aveiro, Portugal

[*] Corresponding author: M. Isabel Cabaço, e-mail: isabelcabaco@tecnico.ulisboa.pt

1. EXPERIMENTAL

1.1. Experimental conditions

The Raman spectra were measured with a resolution of 4 cm^{-1} in the spectral range 200 cm^{-1} to 3800 cm^{-1} on a Horiba Jobin-Yvon XploRA spectrometer using lasers diode operating at a wavelength of 785 nm and 638 nm in a back-scattering geometry. Typical spectra have been collected during 20 seconds and accumulated 60 times to improve the signal-to-noise ratio. In order to take accurate line positions the spectrometer was calibrated by recording different emission lines of a neon bulb.

The Infrared spectra were measured on a Bruker-Alpha FT-IR spectrometer with a 4 cm^{-1} resolution in the spectral range 400 cm^{-1} to 4000 cm^{-1} after collecting 64 scans. We use KBr windows for the absorption Specac Omni-cell and mylar or lead spacers of 6 or 25 micrometer, respectively. The path-lengths have been controlled on the empty cell using the standard method based upon interferences fringes.

X-ray diffraction measurements were performed using a variable geometry device equipped with a Max-Flux TM Optic grade multilayer monochromator for Cu Kα radiation and a gas curved counter INEL CPS 590. It allows obtaining patterns on a range of momentum transfer Q from 0.15 to 2 $Å^{-1}$. Samples were contained in a capillary tube of 1 mm diameter.

1.2. Experimental results

1.2.1. Raman Spectra of the ν_{OH} stretching of TBH corrected from Combination Bands

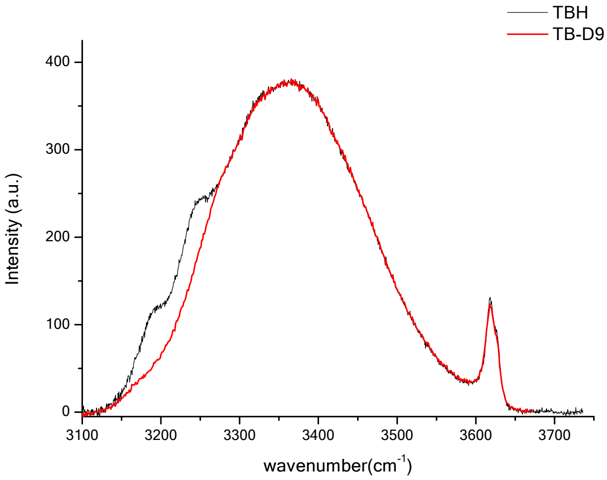

Figure S1. Raman spectra of pure tert-butanol in the spectral domain of ν_{OH}-stretching vibration: hydrogenated (black), tert-butanol-D9 (red).

The Raman spectrum of the ν_{OH} stretching vibration of TBH presents on its low frequency side two bands assigned to combinations vibrations of the CH_3 group. The former, observed

at about 3190 cm^{-1}, involves the symmetric stretching ν_s of the methyl with the out of plane bending vibration τ and the latter, at about 3245 cm^{-1}, the asymmetric stretching vibration ν_a with the τ bending.[1-3] The spectrum of TBH has been corrected from these combination bands using the spectrum of deuterated tert-butanol D9 which is free from these combination transitions (Figure S1)

1.2.2. Dilution of the TBH-TBF mixture in CDCl3

Equimolar TBH – TBF mixtures diluted in $CDCl_3$

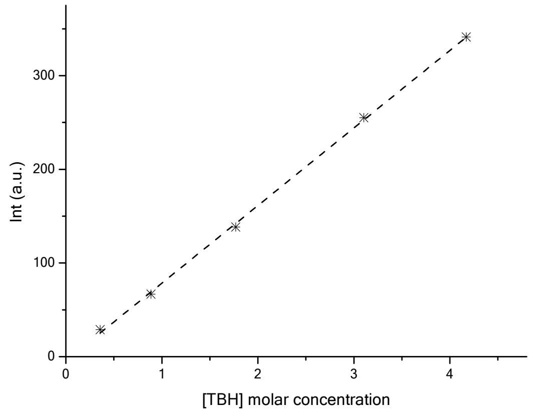

Figure S2. Integrated intensity of the infrared spectra of equimolar TBH – TBF mixtures diluted in $CDCl_3$ measured in the spectral domain 2300-3500 cm^{-1} versus the TBH molar concentration. Intensities are corrected from the C-H stretching contributions (domain 2800 cm^{-1} to 3050 cm^{-1}).

Infrared spectra of TBH – TBF non equimolar mixtures diluted in $CDCl_3$

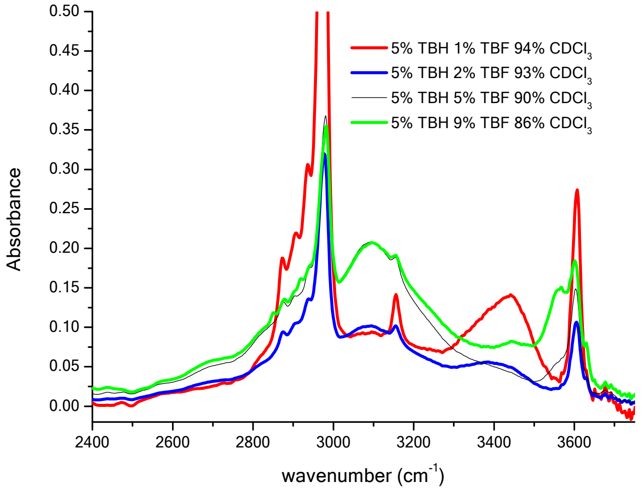

Figure S3. Infrared spectra of TBH – TBF non equimolar mixtures diluted in $CDCl_3$. The spectrum of the diluted equimolar mixture.is displayed for comparison (black).

2. MOLECULAR DYNAMICS SIMULATIONS

2.1. Molecular models

All the intramolecular and dispersive interactions of TBH were modelled as in the original OPLS-AA paper.[4] The model for TBF was adapted from the forcefield of 2,2,2-trifluoroethanol (CF_3CH_2OH) developed by the same research group,[5,6] by replacing each H atom of the CH_2 group with another CF_3 group; the single missing dihedral torsion function (F-C-C-C) was taken from the OPLS-AA model of perfluoroalkanes.[7] The electrostatic interactions of both molecules were modelled by assigning atom-centered partial charges, derived[8] from *ab initio* calculated electron density distributions using the CHELPG method.[9] The full set of atomic partial charges is shown in Table S1. The dispersive Lennard-Jones interactions between different types of atoms were calculated using geometrical mean rules for both size and energy, according to the OPLS framework. To account for the peculiar weak interactions between fluorinated and hydrogenated moieties, the crossed Lennard-Jones parameters between the fluorine atoms of TBF and the methyl hydrogen atoms of TBH were modified from the combining rule values, as proposed for [n-butanol + 2,2,3,3,4,4,4-heptafluor-1-butanol] mixtures in a previous work[10] (cross interaction energy reduced by 20% and cross interaction size increased by 3.5%).

Table S1. Atomic partial charges derived using the CHELPG method from the ab initio electronic density distributions.

TBH		TBF	
atom	charge	atom	charge
C (central)	0.778	C (central)	-0.141
C (in CH_3)	-0.352	C (in CF_3)	0.579
H (in CH_3)	0.071	F	-0.171
O	-0.746	O	-0.479
H (in OH)	0.385	H	0.422

2.2. Simulation details

The simulations were performed using the GROMACS 5.0.7 Software,[11] with systems consisting of either 300 or 3000 total molecules in cubic simulation boxes with periodic

boundary conditions in all directions. A cut-off distance of 14 Å was used for both non-bonded Lennard-Jones and electrostatic potentials, with the application of standard analytic tail corrections for the energy and pressure dispersion terms, and of the particle-mesh Ewald method to the electrostatic interactions beyond the cut-off. A time step of 2 fs was used, with all bonds involving hydrogen atoms constrained to their equilibrium distances using the LINCS algorithm.

All simulations started from random low-density configurations, to which a steepest gradient energy minimization procedure was applied to relax any unphysical high energy contacts between the molecules. A pre-equilibration simulation in the NpT ensemble was performed to each system until its density reached a constant value, using the Berendsen thermostat and barostat with coupling constants of 0.5 and 1 ps, respectively. The systems were then simulated in the NpT ensemble at 298.15 K and 1 bar for at least 2 ns, to determine the equilibrium volume, and the dimensions of the final configuration were rescaled to this value. The simulation results shown were calculated from subsequent NVT trajectories of at least 6 ns, discarding the first 1 ns for equilibration. The Nosé-Hoover thermostat and Parrinello-Rahman barostat were used in the latter runs to control the temperature and pressure, with coupling constants of 0.5 ps and 10.0 ps, respectively.

2.3. Simulation results

Table S2. Probability p'(zz) of finding an aggregate of a given type zz as a function of the TBH–TBF mixture composition, obtained from the molecular dynamics simulations.

$$p'(zz) = \frac{\sum_k (aggregates\ of\ type\ zz)}{\sum_k (all\ aggregates)}$$

a) by size and composition of the aggregate

composition (x(TBH))	1	0.8	0.6	0.5	0.4	0.2	0
H	0.2398	0.1826	0.0757	0.0414	0.0191	0.0040	
F		0.0045	0.0880	0.1907	0.3468	0.6499	0.8524
FF		0.0000	0.0011	0.0050	0.0163	0.0656	0.1263
HF (+ FH)		0.1684	0.4331	0.4941	0.4267	0.1813	
HH	0.1140	0.0584	0.0135	0.0034	0.0011	0.0000	
3F		0.0000	0.0000	0.0001	0.0013	0.0069	0.0193
1H:2F		0.0014	0.0167	0.0424	0.0724	0.0653	
2H:1F		0.1549	0.1935	0.1178	0.0504	0.0050	
3H	0.1256	0.0505	0.0054	0.0010	0.0001	0.0000	
4F		0.0000	0.0000	0.0000	0.0000	0.0006	0.0019
1H:3F		0.0000	0.0003	0.0015	0.0056	0.0096	
2H:2F		0.0054	0.0401	0.0521	0.0423	0.0089	
3H:1F		0.1128	0.0673	0.0228	0.0049	0.0002	
4H	0.2401	0.0665	0.0039	0.0003	0.0000	0.0000	
>4mers	0.2805	0.1946	0.0613	0.0275	0.0131	0.0029	0.0001

b) by size and topology of the aggregate

composition (x(TBH))	1	0.8	0.6	0.5	0.4	0.2	0
monomer	0.2398	0.1871	0.1637	0.2321	0.3659	0.6538	0.8524
dimer	0.1140	0.2268	0.4477	0.5026	0.4440	0.2469	0.1263
lin-3mer	0.0885	0.1854	0.2052	0.1540	0.1182	0.0726	0.0177
cyc-3mer	0.0371	0.0213	0.0104	0.0072	0.0059	0.0045	0.0016
lin-4mer	0.0721	0.1188	0.0898	0.0625	0.0428	0.0160	0.0016
cyc-4mer	0.1464	0.0537	0.0136	0.0077	0.0047	0.0017	0.0002
other-4mer	0.0216	0.0121	0.0083	0.0063	0.0054	0.0017	0.0001
lin-5mer	0.0524	0.0702	0.0341	0.0169	0.0087	0.0023	0.0001
cyc-5mer	0.0169	0.0047	0.0004	0.0002	0.0000	0.0000	0.0000
other-5mer	0.0225	0.0120	0.0045	0.0026	0.0011	0.0002	0.0000
lin-6mer	0.0370	0.0379	0.0113	0.0046	0.0021	0.0003	0.0000
cyc-6mer	0.0047	0.0010	0.0001	0.0000	0.0000	0.0000	0.0000
other-6mer	0.0115	0.0069	0.0022	0.0010	0.0003	0.0000	0.0000
lin-larger	0.1064	0.0478	0.0068	0.0019	0.0007	0.0000	
cyc-larger	0.0011	0.0001	0.0000	0.0000	0.0000	0.0000	

o-larger	0.0280	0.0141	0.0018	0.0003	0.0001	0.0000

3. DFT CALCULATIONS

3.1. DFT Calculations details

The DFT calculations were carried out using the program Gaussian16.C01 package.[12] All the structures of the isolated TBH and TBF molecules (monomers) and of their homo and hetero aggregates are achieved using the Generalized Gradient Approximation (GGA)-hybrid functional B3LYP with the 6-311+G(2d,p) basis set. They were fully optimized using a very tight criterion of energy convergence with superfine integration grids and their vibrational analysis confirmed that all the calculated structures correspond with well-defined local energy minima (no imaginary frequency). Only the aggregates of TBH and/or TBF species having either linear or cyclic as well as open topology have been systematically investigated up to the size of tetramers. Only stable linear, cyclic and open structures are reported below. For higher sized clusters (such as pentamers and hexamers), the topology becomes significantly more sophisticated leading to stable structures, neither linear nor cyclic, but sometimes mixing both (not be reported here). The calculated (interaction) binding energy (ΔE_{int}) were corrected from the basis set superposition error (BSSE).[13-15] In a first step, the vibrational analysis is performed in the harmonic force field approximation in which the harmonic IR and Raman intensities are evaluated for each mode of TBF within a given cluster. In a second step, we have taken into account about the anharmonicity of the ν_{OH}-stretching modes of both TBH and TBF species from their calculated monomer structures using VPT2 method (second order vibrational perturbation theory) implemented in the Gaussian16.C01 software package.[16,17] At this computational level, the anharmonic constants associated with the ν_{OH} stretching mode of both TBH and TBF are evaluated and lead to scaling factor-values (anharmonic/harmonic ratio) about 0.9560 and 0.9525, respectively. For comparison with experimental spectra, these two later scaling factor-values are then used to consider about the anharmonicity of the ν_{OH} stretching modes of both TBH and TBF species within aggregates from the calculated harmonic transitions.

3.2. DFT calculations results

Table S3. BSSE corrected binding energy ΔE^{cor}, Harmonic and scaled vibrational transitions associated with the ν_{OH} and γ_{OH} vibrational modes of TBH molecules in the stable dimers and linear, open and cyclic trimers calculated at the B3LYP/6-311+G(2d,p) level.

	ΔE^{cor} (kcal/mol)	ν_{OH} (cm^{-1})		IR Intensity	IRam activity	*Depolar. ratio ρ*
**H-tBuOH monomer**		**Scaled**	**Harm**	**(km/mol)**	**(Å4/amu)**	
ν_{OH}		3644.4	3812.2	13.9	91.6	0.22
γ_{OH}			284.2	67.8	0.8	0.75
**[H(D)-H(A)] dimer**						
ν_{OH}	-4.5	3495.0<br>3638.1	3655.5<br>3805.1	458.1<br>18.7	240.7<br>68.3	0.26<br>0.18
γ_{OH}			673.6<br>316.5	77.0<br>54.3	1.0<br>1.7	0.48<br>0.75
**[H-H-H]$_L$ lin. trimer**						
ν_{OH}	-10.4	3422.2<br>3460.1<br>3639.9	3579.7<br>3619.3<br>3804.3	525.7<br>584.6<br>18.9	264.6<br>158.9<br>63.7	0.12<br>0.52<br>0.17
γ_{OH}			732.8<br>676.8<br>313.8	16.8<br>71.5<br>58.6	9.0<br>0.3<br>1.0	0.04<br>0.61<br>0.71
**[H-H-H]$_c$ cyc. trimer**						
ν_{OH}	-13.1	3411.4<br>3449.4<br>3468.8	3568.4<br>3608.2<br>3628.5	107.5<br>689.2<br>604.0	380.9<br>117.4<br>107.5	0.11<br>0.60<br>0.54
γ_{OH}			742.0<br>690.4<br>589.4	11.5<br>159.2<br>90.4	11.6<br>0.5<br>0.4	0.03<br>0.62<br>0.58

Table S4. BSSE corrected binding energy ΔE^{cor}, Harmonic and scaled vibrational transitions associated with the ν_{OH} stretching modes of TBH and TBF molecules in the stable dimers and linear, open and cyclic trimers calculated at the B3LYP/6-311+G(2d,p) level.

	ΔE^{cor} (kcal/mol)	ν_{OH} (cm^{-1})		IR Intensity	IRam activity	*Depolar. ratio ρ*
		**Scaled**	**Harm**	**(km/mol)**	**(Å4/amu)**	
**H-tBuOH monomer**		3644.4	3812.2	13.9	91.6	0.22
**F- tBuOH monomer**		3612.6	3792.8	90.3	51.1	0.20
**[H(D)-H(A)] dimer**	-4.5	3495.0<br>3638.1	3655.5<br>3805.1	458.1<br>18.7	240.7<br>68.3	0.26<br>0.18
**[F(D)-F(A)] dimer**	-3.0	3495.6<br>3590.4	3670.1<br>3769.6	574.6<br>122.1	197.7<br>34.3	0.27<br>0.17
**[F(D)-H(A)] dimer**	-9.8	3118.8<br>3637.7	3274.5<br>3804.7	1566.0<br>28.2	208.9<br>61.4	0.34<br>0.15
**$[H\text{-}H\text{-}H]_L$ lin. trimer**	-10.4	3422.2<br>3460.1<br>3639.9	3579.7<br>3619.3<br>3804.3	525.7<br>584.6<br>18.9	264.6<br>158.9<br>63.7	0.12<br>0.52<br>0.17
**$[F\text{-}H\text{-}H]_L$ lin. trimer**	-16.8	2992.0<br>3388.6<br>3638.5	3141.3<br>3544.2<br>3805.5	1654.1<br>711.4<br>21.6	191.1<br>188.8<br>63.0	0.307<br>0.272<br>0.150
**$[F\text{-}F\text{-}H]_L$ lin. trimer**	-14.6	3007.8<br>3441.3<br>3651.0	3157.9<br>3613.1<br>3818.6	1636.9<br>659.7<br>32.0	167.1<br>164.5<br>58.2	0.29<br>0.30<br>0.13
**$[F\text{-}F\text{-}F]_L$ lin. trimer**	-5.5	3436.3<br>3535.1<br>3599.6	3607.7<br>3697.8<br>3765.3	609.3<br>450.3<br>117.9	168.6<br>133.3<br>34.9	0.21<br>0.33<br>0.15
**$[H\text{-}H\text{-}H]_C$ cyclic trimer**	-13.1	3411.4<br>3449.4<br>3468.8	3568.4<br>3608.2<br>3628.5	107.5<br>689.2<br>604.0	380.9<br>117.4<br>107.5	0.11<br>0.60<br>0.54
**$[F\text{-}H\text{-}H]_C$ cyclic trimer**	-17.8	2958.8<br>3403.7<br>3592.2	3105.8<br>3560.0<br>3757.1	1373.4<br>615.3<br>124.1	369.6<br>198.9<br>109.9	0.631<br>0.265<br>0.213
**$[F\text{-}F\text{-}H]_C$ cyc trimer**	-15.0	2998.4<br>3426.0<br>3590.7	3148.0<br>3597.0<br>3755.5	1448.5<br>611.8<br>153.0	197.1<br>155.4<br>113.6	0.29<br>0.27<br>0.21
**$[F\text{-}F\text{-}F]_C$ cyclic trimer**	-8.4	3436.0<br>3466.0<br>3469.1	3607.3<br>3638.8<br>3642.1	8.9<br>739.0<br>718.5	285.6<br>64.1<br>62.0	0.11<br>0.73<br>0.73
**$[FH(D)\text{-}H(A^2)]$ open trimer**	-12.5	3132.1<br>3581.9<br>3624.2	3288.4<br>3746.3<br>3790.6	1382.5<br>159.3<br>27.0	182.1<br>187.0<br>42.5	0.363<br>0.222<br>0.191
**$[FF(D)\text{-}H(A^2)]$ open trimer**	-13.5	3266.7<br>3375.1<br>3607.8	3429.7<br>3543.5<br>3773.4	1053.5<br>717.5<br>35.1	177.4<br>204.2<br>42.2	0.428<br>0.247<br>0.144

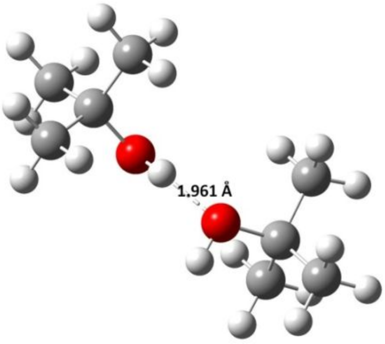

[H-H] Dimer (-4.5 kcal/mol)

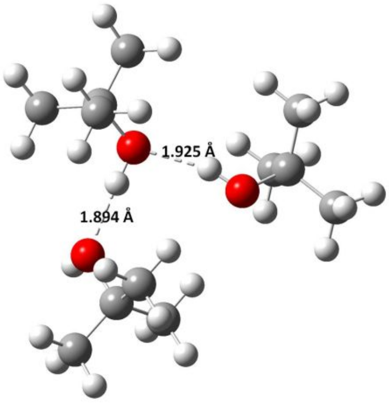

[H-H-H]$_L$-trimer (-5.2 kcal/mol)

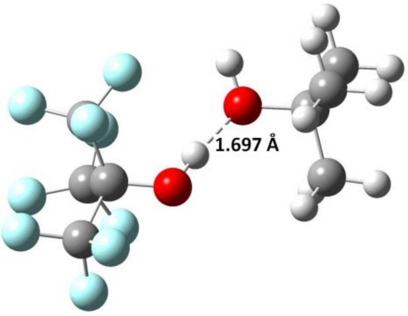

[F-H] Dimer (-9.8 kcal/mol)

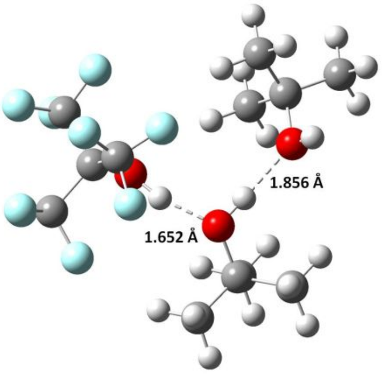

[F-H-H]$_L$-trimer (-8.4 kcal/mol)

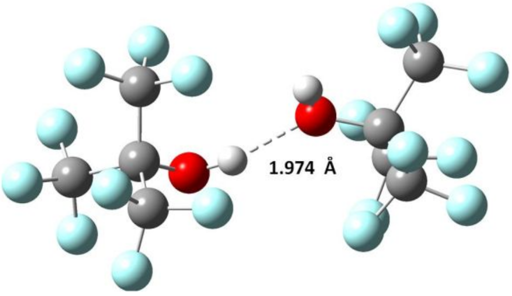

[F-F] Dimer (-3.0 kcal/mol)

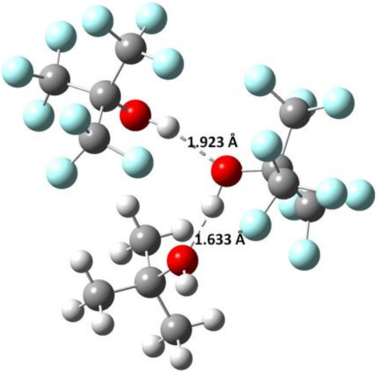

[F-F-H]$_L$-trimer (-7.5 kcal/mol)

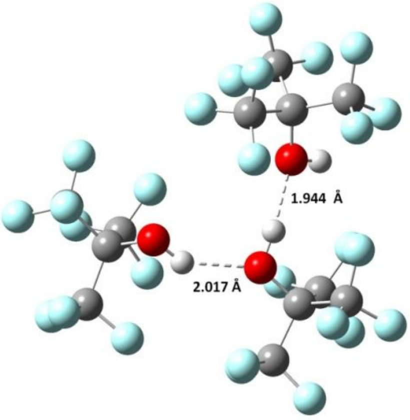

[F-F-F]$_L$-trimer (-2.8 kcal/mol)

Figure S4. Calculated dimer and linear trimer structures [B3LYP/6-311+G(2d,p) level]. Energy-values in parentheses are the BSSE corrected Binding Energies per bond.

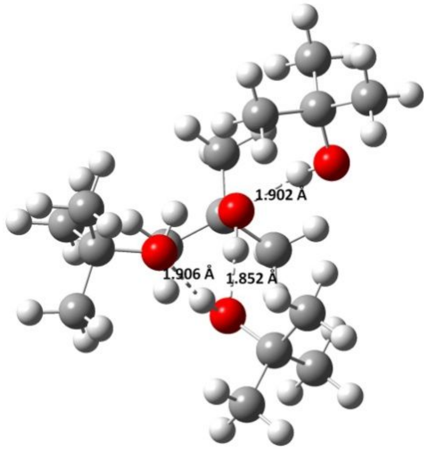

[H-H-H]$_c$-trimer (-4.4 kcal/mol)

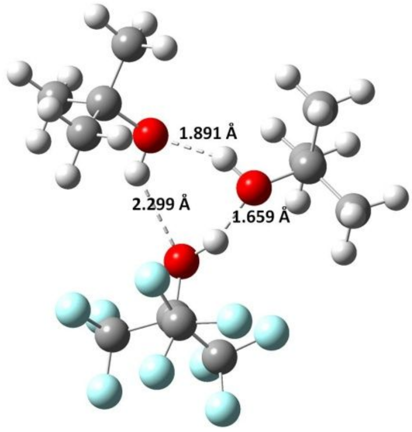

[F-H-H]$_c$-trimer (-5.7 kcal/mol)

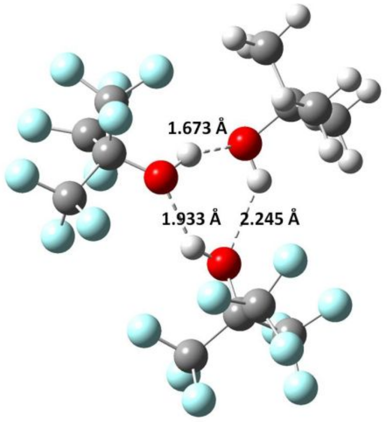

[F-F-H]$_c$-trimer (-5.0 kcal/mol)

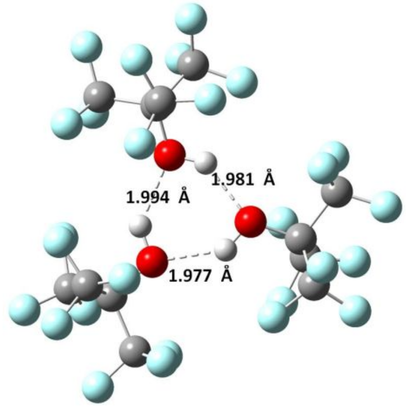

[F-F-F]$_c$-trimer) (-2.8 kcal/mol)

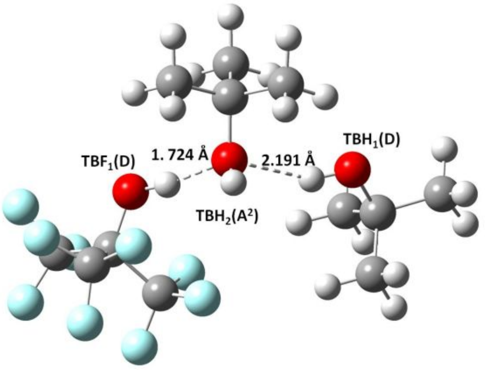

[FH(D)-H(A^2)]open trimer(-6.3 kcal/mol)

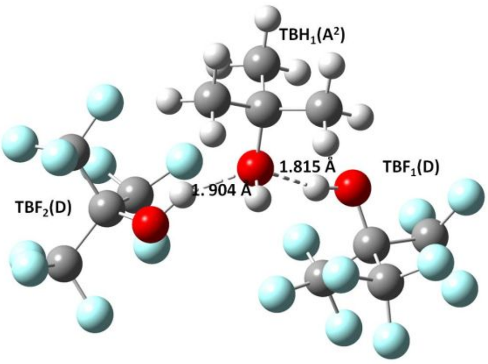

[FF(D)-H(A^2)]open trimer (-6.8 kcal/mol)

Figure S5. Calculated open and cyclic trimer structures [B3LYP/6-311+G(2d,p) level]. Energy-values in parentheses are the BSSE corrected Binding Energies per bond.

Table S5. Idem Table S4. for stable tetramers [B3LYP/6-311+G(2d,p) level].

	ΔE^{cor} (kcal/mol)	ν_{OH} (cm^{-1})		IR Intensity	IRam activity	*Depolar. ratio ρ*
		**Scaled**	**Harm**	**(km/mol)**	**(Å4/amu)**	
**[H-H-H-H]$_L$ tetramer**	-16.0	3348.4<br>3336.9<br>3444.2<br>3620.1	3502.5<br>3595.1<br>3602.7<br>3786.7	653.2<br>582.4<br>520.0<br>77.2	242.7<br>161.6<br>177.9<br>138.1	0.11<br>0.46<br>0.263<br>0.246
**[F-H-H-H]$_L$ tetramer**	-24.0	2939.2<br>3283.4<br>3416.7<br>3630.9	3085.8<br>3434.5<br>3574.0<br>3798.0	921.3<br>871.9<br>602.0<br>91.3	96.4<br>190.7<br>176.6<br>102.0	0.227<br>0.223<br>0.312<br>0.211
**[F-F-H-H]$_L$ tetramer**	-21.9	2779.1<br>3351.1<br>3418.1<br>3632.9	2917.7<br>3518.2<br>3575.4<br>3800.1	1992.1<br>740.4<br>695.8<br>32.5	115.2<br>178.0<br>148.0<br>61.2	0.330<br>0.243<br>0.315<br>0.153
**[F-H-F-H]$_L$ tetramer**	-22.9	3000.9<br>3037.2<br>3555.8<br>3636.6	3150.5<br>3188.7<br>3719.0<br>3803.5	1581.7<br>1552.7<br>270.1<br>30.9	172.9<br>170.0<br>145.2<br>57.5	0.234<br>0.396<br>0.232<br>0.149
**[F-F-F-H]$_L$ tetramer**	-17.9	2925.7<br>3368.6<br>3490.8<br>3639.9	3071.6<br>3536.6<br>3664.9<br>3807.4	1719.2<br>736.15<br>497.6<br>31.0	135.4<br>153.0<br>126.0<br>58.4	0.221<br>0.247<br>0.312<br>0.143
**[F-F-F-F]$_L$ tetramer**	-8.3	3428.0<br>3464.8<br>3507.6<br>3558.8	3598.9<br>3637.6<br>3682.5<br>3736.3	467.4<br>530.7<br>433.8<br>283.5	173.1<br>99.0<br>131.8<br>62.1	0.109<br>0.419<br>0.347<br>0.362
**[H-H-H-H]$_C$ tetramer**	-23.1	3280.6<br>3335.7<br>3335.9<br>3362.3	3431.6<br>3489.2<br>3489.4<br>3517.0	0.<br>1306.5<br>1306.7<br>326.3	459.0<br>55.9<br>55.9<br>179.9	0.049<br>0.75<br>0.75<br>0.75
**[F-H-H-H]$_C$ tetramer**	-26.2	2775.8<br>3234.0<br>3382.9<br>3560.7	2914.2<br>3382.8<br>3538.6<br>3724.6	1917.3<br>954.9<br>684.9<br>262.8	128.2<br>187.9<br>164.1<br>136.7	0.314<br>0.218<br>0.295<br>0.240
**[F-F-H-H]$_C$ tetramer**	-24.3	2675.7<br>3312.2<br>3380.2<br>3591.9	2809.1<br>3477.4<br>3535.8<br>3757.2	2060.6<br>736.7<br>797.5<br>186.5	113.9<br>185.9<br>139.6<br>122.4	0.313<br>0231<br>0.298<br>0.231
**[F-H-F-H]$_C$ tetramer**	-26.2	2879.8<br>2994.3<br>3535.2<br>3543.6	3023.5<br>3143.7<br>3697.5<br>3706.3	1405.0<br>1723.8<br>256.0<br>389.9	67.8<br>151.1<br>182.7<br>114.5	0.678<br>0.389<br>0.193<br>0.293
**[F-F-F-H]$_C$ tetramer**	-19.7	2925.0<br>3328.4<br>3469.7<br>3582.6	3070.9<br>3494.4<br>3642.7<br>3747.5	1608.0<br>820.15<br>482.4<br>277.8	156.4<br>155.7<br>120.2<br>127.7	0.191<br>0.257<br>0.257<br>0.231
**[F-F-F-F]$_C$ tetramer**	-12.1	3404.7<br>3433.5<br>3428.4<br>3450.4	3561.4<br>3591.5<br>3599.4<br>3622.5	73.6<br>886.6<br>786.5<br>577.3	275.2<br>73.7<br>82.8<br>87.9	0.175<br>0.692<br>0.617<br>0.6615
**[FF(D)-H(AD)H(A^2)] open tetramer**	-21.3	3120.9<br>3209.5<br>3514.9<br>3615.2	3276.7<br>3369.7<br>3676.3<br>3781.2	1335.2<br>1131.2<br>291.1<br>22.9	165.0<br>183.5<br>18.2<br>177.6	0.444<br>0.220<br>0.100<br>0.228

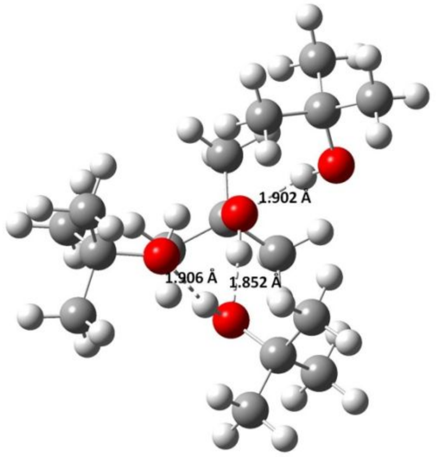

[H-H-H-H]$_L$-tetramer (-5.2 kcal/mol)

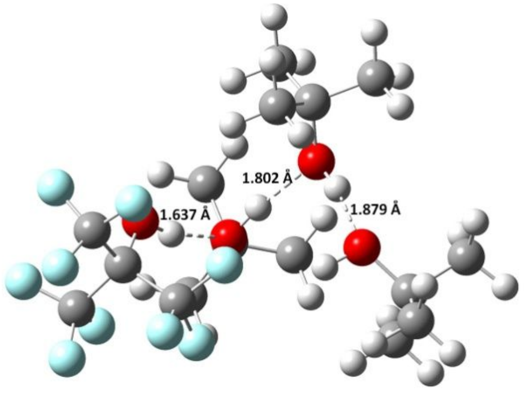

[F-H-H-H]$_L$-tetramer (-8.0 kcal/mol)

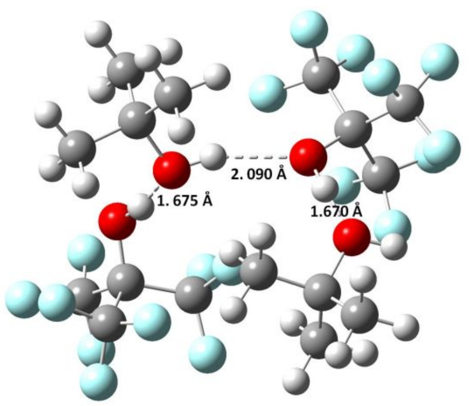

[F-H-F-H]$_L$-tetramer (-7.6 kcal/mol)

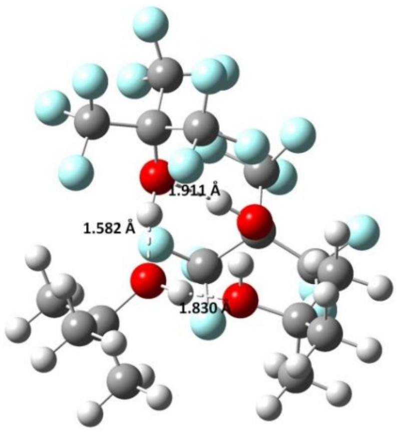

[F-F-H-H]$_L$-tetramer(-7.3 kcal/mol)

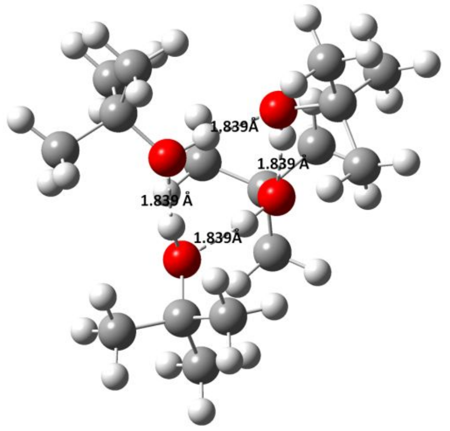

[H-H-H-H]$_c$-tetramer (-5.8 kcal/mol)

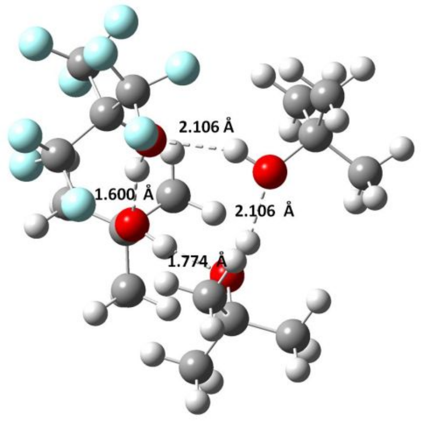

[F-H-H-H]$_c$ -tetramer (-6.7 kcal/mol)

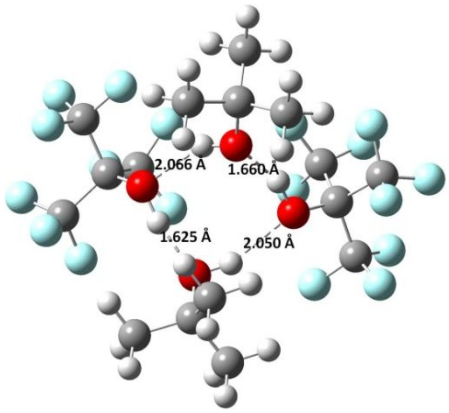

[F-H-F-H]$_c$-tetramer (-6.5 kcal/mol)

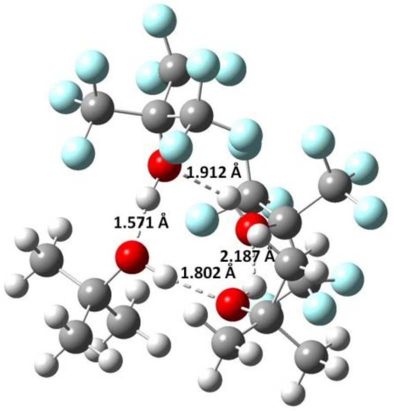

[F-H-F-H]$_c$-tetramer (-6.1 kcal/mol)

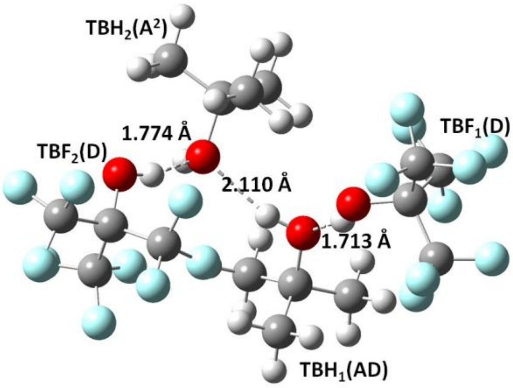

[FF(D)-H(AD)H(A^2)] open tetramer (-7.1 kcal/mol)

Figure S6. Calculated open, linear and cyclic tetramer structures [B3LYP/6-311+G(2d,p) level].Energy-values in parentheses are the BSSE corrected Binding Energies per bond.

Table S6. Harmonic and anharmonic vibrational transitions, overtones and combinations bands, associated with the $\mathbf{\nu_{OH}}$ and $\mathbf{\nu_{CO}}$ stretching and $\mathbf{\delta_{COH}}$ bending modes of TBH and TBF in monomer structure and in stable dimer calculated at the B3LYP/6-311+G(2d,p) level.

		Harm		AnHarm		
		**ν-transition (cm^{-1})**	**I_{IR} (km/mol)**	**ν-transition (cm^{-1})**	**I_{IR} (km/mol)**	**modes**
**[H-terBuOH]**						
d_{O-H} (Å)	0.9643	3812.2	13.9	3644.9	11.8	ν_{OH}
d_{C-O} (Å)	1.4427	1228.5	48.8	1198.8	42.1	ν_{CO}
$\alpha_{C.O-H}$	108.594	1360.8	32.4	1320.6	26.2	δ_{COH}
		284.2	67.8	439.3	2.3	γ_{OH}
overtones				2635.6	0.2	$2\delta_{COH}$
				2394.2	~0.	$2\nu_{CO}$
**[F-terBuOH]**						
d_{O-H} (Å)	0.9671	3792.8	90.3	3612.5	83.3	ν_{OH}
d_{C-O} (Å)	1.3909	1160.0	134.4	1135.9	286.9	ν_{CO}
$\alpha_{C.O-H}$	109.798	1387.7	59.6	1352.8	35.5	δ_{COH}
		283.5	37.5	235.3	33.5	γ_{OH}
overtones				2690.7	0.7	$2\delta_{COH}$
				2259.5	~0.	$2\nu_{CO}$
**[F,H]-terBuOH Dimer**						
$d_{O-H(A)}$ (Å)	0.9654	3804.7	28.2	3624.9	23.1	$\nu_{OH(A)}$
$d_{O-H(D)}$ (Å)	0.9927	3274.5	1566.1	3034.1	641.0	$\nu_{OH(D)}$
$d_{(CF3)_3C-O}$(Å)	1.3773	1290.9	47.2	1268.3	32.3	**ν_{CO}(TBF)**
$d_{(CH3)_3C-O}$(Å)	1.4617	1217.9	55.5	1187.2	17.7	**ν_{CO}(TBH)**
$\alpha_{(CF3)_3C-O-H}$	112.910	1480.0	105.0	1417.7	0.5	**δ_{COH}(TBF)**
$\alpha_{(CH3)_3C-O-H}$	109.117	1360.8	22.5	1316.2	11.2	**δ_{COH}(TBH)**
		797.1	98.6	678.2	2.6	**γ_{OH}(TBF)**
		316.5	1.0	308.2	26.7	**γ_{OH}(TBH)**
overtones						
				2837.2	80.2	**$2\delta_{COH}$(TBF)**
				2626.2	0.3	**$2\delta_{COH}$(TBH)**
Combination bands				2695.7	7.0	$\nu_{CO(D)}\otimes\delta_{COH(D)}$
				2833.1	4.3	$\{\delta_{CH_3}\}\otimes\delta_{COH(D)}$
				2853.5	45.3	idem
				2881.7	8.0	idem
				2887.0	42.3	idem
				2898.3	5.2	idem
				2911.9	2.0	idem

4. Description of the IR broad band observed in equimolar mixture TBH-TBF

Table S7. DFT scaled band centre frequency and infrared activity. The probability of found an aggregate in the equimolar mixture TBH-TBF was obtained from MD (Table S2.). The integrated intensity associated to each aggregate was calculated multiplying its IR activity by its probability. The widths (FWHH) of the adjusted lorentzian profiles to the experimental profile are presented.

Aggregate	Scaled frequency (cm^{-1})	IR activity (km mol^{-1})	Probability	FWHH (cm^{-1})
H	3645	14	0.0414	15
F	3612	90	0.1907	40
HH (D)	3495	458	0.0034	70
HH (A)	3638	19		30
FF (D)	3496	575	0.005	70
FF (A)	3590	122		30
FH (D)	3119	1566	0.4941	260
FH (A)	3638	28		15
FHH Lin	2992	1654	0.1119	290
FHH Lin	3389	711		200
FHH Lin	3639	22		30
FHH cyc	2959	1373	0.0059	290
FHH cyc	3404	615		200
FHH cyc	3592	124		70
FFH Lin	3008	1637	0.0403	290
FFH Lin	3441	660		200
FFH Lin	3651	32		30
FFH cyc	2998	1449	0.0021	290
FFH cyc	3426	612		200
FFH cyc	3591	153		70
FHFH lin	3001	1582	0.0427	290
FHFH lin	3037	1553		290
FHFH lin	3556	270		30
FHFH lin	3637	31		30
FHFH cyc	2880	1405	0.0052	290
FHFH cyc	2994	1724		290
FHFH cyc	3535	256		50
FHFH cyc	3544	390		50
FHFH open	3121	1335	0.0042	290
FHFH open	3210	1131		290
FHFH open	3515	291		30
FHFH open	3615	23		30
FHHH lin	2939	921	0.0187	290
FHHH lin	3284	872		290
FHHH lin	3417	602		200
FHHH lin	3631	91		30
FHHH cyc	2776	1917	0.0023	290
FHHH cyc	3234	955		290
FHHH cyc	3383	685		200
FHHH cyc	3561	263		70

References

1. Korppi-Tommola, J. Tert-Butyl Alcohol – Matrix I.R. Spectra and Vibration Assignment. *Spectrochim. Acta, Part A* **1978**, *34*, 1077-1085.
2. Sassi, P.; Palombo, F.; Cataliotti, R. S.; Paolantoni, M.; Morresi, A. Distributions of H-Bonding Aggregates in Tert-Butyl Alcohol: The Pure Liquid and Its Alkane Mixtures. *J. Phys. Chem. A* **2007**, *111*, 6020-6027.
3. Abdel Hamid, A. R.; Lefort, R.; Lechaux, Y.; Moréac, A.; Ghoufi, A.; Alba-Simionesco, C.; Morineau, D. Solvation Effects on Self-Association and Segregation Processes in Tert-Butanol–Aprotic Solvent Binary Mixtures. *J. Phys. Chem. B* **2013**, *117*, 10221-10230.
4. Jorgensen, W.L.; Maxwell, D.S.; Tirado-Rives, J. Development and Testing of the OPLS All-Atom Force Field on Conformational Energetics and Properties of Organic Liquids. *J. Am. Chem. Soc.* **1996**, *118*, 11225–11236.
5. Duffy, E. M. Targeting Key Recognition Elements in Bioorganic Systems. Ph.D. Dissertation, Yale University, 1994.
6. Chitra, R.; Smith, P. E. A Comparison of the Properties of 2,2,2- Trifluoroethanol and 2,2,2-Trifluoroethanol/Water Mixtures Using Different Force Fields. *J. Chem. Phys.* **2001**, *115*, 5521−5530.
7 Watkins, E. K.; Jorgensen, W. L. Perfluoroalkanes: Conformational Analysis and Liquid-State Properties from Ab Initio and Monte Carlo Calculations. *J. Phys. Chem. A* **2001**, *105*, 4118−4125.
8. Cabaço, M. I.; Besnard, M.; Cruz, C.; Morgado, P.; Silva, G. M. C.; Filipe, E. J. M.; Coutinho, J. A. P.; Danten, Y. The Structure of Liquid Perfluoro Tert-Butanol Using Infrared, Raman and X-Ray Scattering Analyzed by Quantum DFT Calculations and Molecular Dynamics. *Chem. Phys. Lett.* **2021**, *779*, 138844.
9. Breneman, C. M.; Wiberg, K.B. Determining Atom-centered Monopoles from Molecular Electrostatic Potentials. The Need for High Sampling Density in Formamide Conformational Analysis. *J. Comput. Chem.* **1990**, *11*, 361-373.
10. Morgado, P.; Garcia, A.R.; Ilharco, L.M.; Marcos, J.; Anastácio, J.; Martins, L. F. G.; Filipe, E. J. M. Liquid Mixtures Involving Hydrogenated and Fluorinated Alcohols: Thermodynamics, Spectroscopy, and Simulation. *J. Phys. Chem. B.* **2016,** *120*, 10091–10105.
11. Van Der Spoel, D.; Lindahl, E.; Hess, B.; Groenhof, G.; Mark, A. E.; Berendsen, H. J. C. GROMACS: Fast, Flexible, and Free. *J. Comput. Chem.* **2005**, *26*, 1701-1718.
12. Frisch, M. J.; Trucks, G. W.; Schlegel, H. B.; Scuseria, G. E.; Robb, M. A.; Cheeseman, J. R.; Scalmani, G.; Barone, V.; Petersson, G. A.; Nakatsuji, H. et al. *Gaussian 16*, Revision C.01, Gaussian, Inc., Wallingford CT, 2016.
13. Boys, S. F.; Bernardi, F. The Calculation of Small Molecular Interactions by the Differences of Separate Total Energies. Some Procedures with Reduced Errors. *Mol. Phys*. **1970**, *19*, 553-566.
14. Valiron, P.; Mayer, I. Hierarchy of Counterpoise Corrections for *N*-body Clusters: Generalization of the Boys-Bernardi Scheme. *Chem. Phys. Lett.* **1997**, *275*, 46-55.
15. White, J. C.; Davidson, E. R. An Analysis of the Hydrogen Bond in Ice. *J. Chem. Phys.* **1990**, *93*, 8029-8035.
16. Barone, V. Anharmonic Vibrational Properties by a Fully Automated Second-order Perturbative Approach. *J. Chem. Phys.* **2005**, *122*, 014108.
17. Barone, V. Vibrational Zero-Point Energies and Thermodynamic Functions Beyond the Harmonic Approximation. *J. Chem. Phys.* **2004**, *120*, 3059-3065.
